# Serological Responses of Raccoons and Striped Skunks to Ontario Rabies Vaccine Bait in West Virginia during 2012–2016

**DOI:** 10.3390/v13020157

**Published:** 2021-01-22

**Authors:** Shylo R. Johnson, Dennis Slate, Kathleen M. Nelson, Amy J. Davis, Samual A. Mills, John T. Forbes, Kurt C. VerCauteren, Amy T. Gilbert, Richard B. Chipman

**Affiliations:** 1 USDA/APHIS/WS/National Wildlife Research Center, 4101 LaPorte Ave., Fort Collins, CO 80521, USA; amy.j.davis@usda.gov (A.J.D.); kurt.c.vercauteren@usda.gov (K.C.V.); amy.t.gilbert@usda.gov (A.T.G.); 2 USDA/APHIS/WS/National Rabies Management Program, 59 Chenell Dr., Concord, NH 03301, USA; dslate@outlook.com (D.S.); kathleen.m.nelson@usda.gov (K.M.N.); richard.b.chipman@usda.gov (R.B.C.); 3 USDA/APHIS/Wildlife Services, 730 Yokum St., Elkins, WV 26241, USA; samual.a.mills@usda.gov (S.A.M.); john.forbes@usda.gov (J.T.F.)

**Keywords:** bait density, ONRAB, oral rabies vaccination, rabies virus, raccoon, skunk, virus-neutralizing antibody

## Abstract

Since the 1990s, oral rabies vaccination (ORV) has been used successfully to halt the westward spread of the raccoon rabies virus (RV) variant from the eastern continental USA. Elimination of raccoon RV from the eastern USA has proven challenging across targeted raccoon (*Procyon lotor*) and striped skunk (*Mephitis mephitis*) populations impacted by raccoon RV. Field trial evaluations of the Ontario Rabies Vaccine Bait (ONRAB) were initiated to expand ORV products available to meet the rabies management goal of raccoon RV elimination. This study describes the continuation of a 2011 trial in West Virginia. Our objective was to evaluate raccoon and skunk response to ORV occurring in West Virginia for an additional two years (2012–2013) at 75 baits/km^2^ followed by three years (2014–2016) of evaluation at 300 baits/km^2^. We measured the change in rabies virus-neutralizing antibody (RVNA) seroprevalence in targeted wildlife populations by comparing levels pre- and post-ORV during each year of study. The increase in bait density from 75/km^2^ to 300/km^2^ corresponded to an increase in average post-ORV seroprevalence for raccoon and skunk populations. Raccoon population RVNA levels increased from 53% (300/565, 95% CI: 50–57%) to 82.0% (596/727, 95% CI: 79–85%) during this study, and skunk population RVNA levels increased from 11% (8/72, 95% CI: 6–20%) to 39% (51/130, 95% CI: 31–48%). The RVNA seroprevalence pre-ORV demonstrated an increasing trend across study years for both bait densities and species, indicating that multiple years of ORV may be necessary to achieve and maintain RVNA seroprevalence in target wildlife populations for the control and elimination of raccoon RV in the eastern USA.

## 1. Introduction

Oral rabies vaccination (ORV) is a proven prevention and control method for large-scale landscape applications targeting rabies viruses (RV) circulating in wildlife [1]. ORV has been successfully used to eliminate the canine RV variant in coyotes (*Canis latrans*) in south Texas, USA [2,3] and RV in red foxes (*Vulpes vulpes*) and raccoon dogs (*Nyctereutes procyonoides*) across Europe [4]. The use of ORV targeting red foxes and striped skunks (*Mephitis mephitis*) eliminated the arctic fox (*Vulpes lagopus*) RV from large areas in southern Ontario, Canada [5,6]. However, related viruses have re-emerged in red foxes and striped skunks in southwestern Ontario, requiring new management activities for arctic fox RV control [7]. Strategies focused on ORV have proven effective across species and geographic areas and have prevented the westward spread of raccoon RV from the eastern USA.

Rabies was first reported in raccoons (*Procyon lotor*) in Florida during the 1940s [8]. Raccoon RV spread from Florida north into South Carolina and west into Alabama during the 1960s and 1970s [9]. An unintentional translocation of infected raccoons in 1977 from Florida to West Virginia resulted in a RV epizootic that rapidly spread along the USA eastern seaboard [10,11] and raccoon RV remains enzootic throughout the eastern seaboard of the USA [12]; consequently, much of this region faces higher disease prevention costs due to higher risks of rabies exposure from raccoons, skunks and other animals infected with raccoon RV [13,14,15]. During 1998, the United States Department of Agriculture, Animal and Plant Health Inspection Service, Wildlife Services, National Rabies Management Program (NRMP) received its first Congressional appropriation to prevent terrestrial RV spread [16] and established the national goal to eliminate specific wildlife RVs in the USA. Critical to any wildlife RV elimination effort is achieving adequate population immunity to reduce the susceptible fraction of hosts and prevent RV transmission among reservoir populations. Meso-carnivore wildlife reservoirs implicated in the routine spillover of RV can leverage a strong impact on the success of control efforts, e.g., the expanded ORV management focus across Europe formalized in 2015 to include raccoon dogs as targeted vectors of red fox RV [17]. ORV campaigns in the USA have varied in effectiveness as measured by post-bait RV neutralizing antibody (RVNA) seroprevalence [15,18], yet estimates have also been developed to estimate prevalence reduction among target populations [19,20].

Prior to 2011, the only vaccine available for use in the USA was RABORAL V-RG^®^ (Boehringer Ingelheim [formerly Merial, Inc.], Duluth, GA, USA), which has been used to control RV in coyotes and raccoons and also used experimentally to target gray foxes (*Urocyon cinereoargenteus*) and striped skunks [21]. The RVNA seroprevalence achieved using RABORAL V-RG has averaged 30% among orally vaccinated raccoon populations [22], and is estimated to be lower (3–7%) among orally vaccinated skunk populations in the eastern USA [15,18]. With a goal of raccoon RV elimination, the NRMP has an interest to evaluate and refine ORV products and strategies for striped skunks, which are key spillover hosts of raccoon RV [23,24]. Skunks have a history of being spillover hosts to heterologous wildlife RV, as occurred in Arizona with a bat RV [25] and arctic fox RV in southeastern Ontario [6]. Moreover, there is concern that raccoon RV may persist in striped skunk populations and compromise local raccoon RV elimination success in areas of the eastern USA [23,24].

The Ontario Rabies Vaccine Bait (ONRAB, Artemis Technologies, Inc. Guelph, Ontario, Canada) has been a prospective ORV tool for raccoon RV elimination based on post-distribution RVNA seroprevalence levels observed in raccoon and striped skunk populations in Canada and the USA [5,26,27,28,29]. The ONRAB bait contains the vaccine in a blister pack coated with a sweet bait matrix containing 100 mg of the biomarker tetracycline hydrochloride (TTCC; see Graham and Prevec [30], Rosatte et al. [31]). During 2011, the first ONRAB field trial in the USA occurred in an ORV-naïve area with enzootic raccoon RV in southeastern West Virginia. That application resulted in one of the highest RVNA seroprevalence levels among raccoons after ORV baiting in an ORV-naïve area of the eastern USA [32]. We discuss the results from a continued evaluation in West Virginia for two consecutive years (2012–2013) at a density of 75 baits/km^2^ followed by three consecutive years (2014–2016) of ORV using ONRAB at 300 baits/km^2^. Our objective was to evaluate the impact of ORV using standard and high-density baiting methods by measuring and comparing the RVNA responses among targeted raccoon and striped skunk populations in a rural area of West Virginia.

## 2. Materials and Methods

### 2.1. Study Area and Design

**Figure 1:**
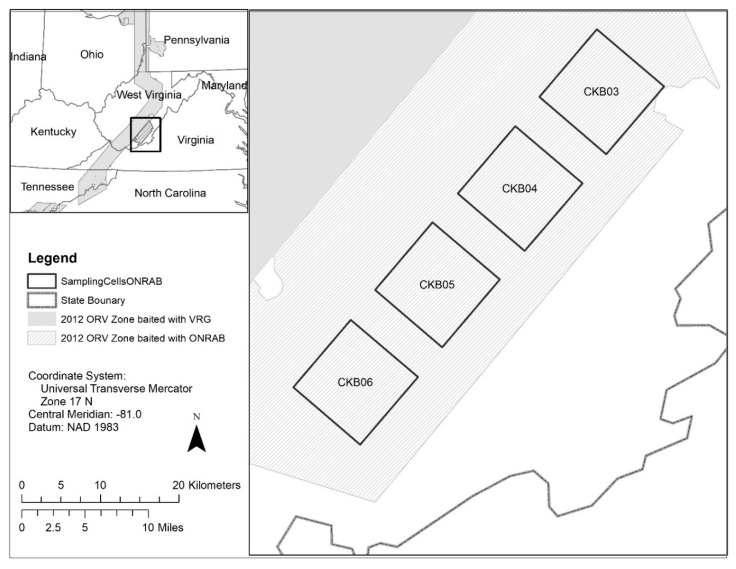
Location of the cells sampled during the 2012–2016 oral rabies vaccination (ORV) field trials in West Virginia, USA. Four cells (CKB03, CKB04, CKB05, and CKB06) within the Ontario Rabies Vaccine Bait (ONRAB) field trial area were sampled during the period 2012–2013 when the area was baited at a target of 75 baits/km^2^. Three cells (CKB03, CKB04, CKB05) continued to be sampled during the period 2014–2016 when the area was baited with ONRAB at a target of 300 baits/km^2^.

The 2012–2013 ONRAB field trial was conducted in West Virginia, USA (37 47′ N, 80 37′ W) as a continuation of a study initiated during 2011 [32]. Raccoon and striped skunk marking and sampling occurred within four 127 km^2^ cells established during 2011 that were separated by 5 km buffers and located to minimize edge effects around areas where target wildlife were sampled. Study cells were located in Monroe, Summers, and Greenbrier Counties and consisted mainly of forest and agriculture habitat (Figure 1). The ONRAB baits were distributed at 75/km^2^ with 750 m parallel flight lines or by ground means (hand distribution from vehicles) throughout this area similar to 2011 (see Slate et al. [32]). During the period 2014–2016, we reduced pre- and post-ORV sampling to three of the four original cells. Target bait density was quadrupled to 300/km^2^ and flight-line spacing was reduced to 250 m, similar to ORV strategies reported by Rosatte et al. [5].

We grouped habitat into five categories based on the National Land Cover Database (NCLD)—agriculture (values 71, 81, 82), forested (values 41, 42, 43, 52), developed (values 21, 22, 23, 24), water (values 11, 90, 95), and barren (value 31)—and habitat composition was based on the 2011 NCLD [33] for the four cells sampled during the period 2012–2013, which were 65% forested, 28% agriculture, 6% developed, 1% water and <1% barren. For the three cells sampled during the period 2014–2016, the habitat composition was based on the 2016 NLCD [34] and the cells were 63% forested, 30% agriculture, 7% developed, 1% water and <1% other.

### 2.2. Animal Sampling

A random point system was used to guide trap placement of 150 traps within cells, as described by Slate et al. [32] and Gilbert et al. [28]. Trapping occurred during July and August prior to ORV and during October post-ORV (Table 1). Methods for generating points were the same as described by Gilbert et al. [28]. The same points were used for the two trapping periods within each year and new points were generated each consecutive year. For a single cell, each pre- and post-ORV trapping period consisted of 10 consecutive nights. During 2014 and 2015, skunk-focused trapping was used to complement the random location trapping efforts to increase sample sizes. Skunk-focused trapping was accomplished by trapping on cell properties with a history of skunk presence based on landowner reports and past trapping experience. The number of trap nights for enhanced skunk trapping varied based on capture success on the properties, which ranged from 1 to 44 traps per night. Skunk-focused trapping during 2014 occurred for 17 days and 13 days in the pre- and post-ORV periods, respectively, and during 2015 for 12 days and 13 days in the pre- and post-ORV periods, respectively. This study was conducted in compliance with the National Wildlife Research Center (NWRC) Institutional Care and Use Committee (IACUC) guidelines (protocols QA-1905, 2028, and 2313). The skunk focused trapping in the period 2014–2015 occurred under the approval of the NWRC IACUC. Standard live-trapping and sampling were IACUC deferred due to the activities described being considered as routine management.

All live trapping occurred using cage traps (Tomahawk Live Trap, LLC, Hazelhurst, WI, USA). Animals with abnormal behavior or suspect lesions were euthanized and tested by a direct rapid immunohistochemistry test (dRIT) [35]. The dRIT positives were sent to the Centers for Disease Control and Prevention (CDC) for diagnostic confirmation and RV variant typing. Captured and apparently healthy target species (i.e., raccoons, striped skunks, red foxes, gray foxes, and coyotes) were anesthetized using a 5:1 mixture of ketamine:xylazine for body measurements and sample collection, whereas nontarget animals were immediately released from traps at the location of capture. For each target animal, we determined sex, relative age (adult or juvenile), and weight (kg); we collected a blood sample from a peripheral vein, extracted a first premolar tooth, and applied an unique numbered ear tag to both ears (National Band and Tag Company, Newport, KY, USA). Target species were processed once per trapping period, but individuals were re-processed when recaptured during a subsequent trapping period. Healthy appearing target animals were released at the point of capture after recovery from anesthesia. On the day of capture, serum samples were separated from whole blood by centrifugation and aliquots were stored in labeled cryovials at −25 to −70 °C until analysis.

### 2.3. RVNA Determination

Sera were analyzed using a modified neutralization test [36] by the New York State Department of Health (NYSDOH). Similar to Gilbert et al. [28], we report main results using a cutoff of 0.125 IU/mL to identify RVNA seropositive animals. We also present results using a higher 0.5 IU/mL cutoff (Supplementary Files, Figures S1 and S3, Tables S4, S5, S11, S13 and S15–S19). Samples with RVNA values less than the cutoff were considered seronegative.

A subset of samples was independently analyzed using a standard rapid fluorescent focus inhibition test [37] at Kansas State University. The subset consisted of 300 raccoon sera and 39 skunk sera that tested within the range of 0.125–0.5 IU/mL at NYSDOH (Table S1).

### 2.4. Tetracycline Biomarker and Age Determination

Teeth were processed for TTCC deposition and age class at Matson’s Laboratory (Manhattan, Montana, USA). Levels of TTCC deposition were determined with a Leitz compound microscope (Leica Microsystems GmbH, Wetzlar, Germany) using ultraviolet light at 100× magnification. Records from 626 (21%) raccoons and 86 (30%) skunks were missing TTCC results due to either a poor-quality tooth sample or no tooth sample available. Age determination was made by cementum annuli counts [38]. In the field, relative age for juvenile (<1 year) and adult (≥1 year) was assessed from animal body size, weight, and reproductive status [39]. A specific year age class assignment was not possible for 659 (22%) raccoon and 98 (34%) skunk records due to either a poor-quality tooth sample or no tooth sample available.

### 2.5. Statistical Methods

We used a generalized linear mixed model (GLMM) implemented in program R [40] to compare seroprevalence responses in raccoon or skunk populations under the ORV conditions applied, which include bait density (75/km^2^ or 300/km^2^), duration of baiting in years (at a specific density), and sampling period (pre- or post-ORV). We also investigated the interaction between baiting duration and sampling period, to account for trends over time pre-ORV compared to post-ORV. Seroprevalence was modeled with a beta distribution which ranges between zero and one (*betareg*) [41]. We treated study cell as a random effect term to account for spatial covariance structure in the response data from populations sampled. We repeated this analysis using TTCC results as the response variable (Supplementary File, Figure S8 and Table S10). We also conducted a model selection on seroprevalence to compare the weights and ranks in fit to the study data (Supplementary File and Tables S11–S15).

We used generalized additive modeling (GAM) to examine seroprevalence at the individual animal level. Analysis occurred using the package *mgcv* (v 1.8-28) [42]. Raccoon and skunk analyses were conducted separately and the same set of models were compared within each species using Akaike’s information criterion corrected (AICc) for small sample sizes [43]. We tested the same models (Table S2) using three different binary response variables: RVNA at the 0.125 IU/mL cutoff, RVNA at the 0.5 IU/mL cutoff (Supplementary File and Tables S16–S19), and binary TTCC detection (Supplementary File and Tables S20–S23). Our predictor variables were animal age (years), sex (male or female), period (pre- or post-ORV), bait density (75/km^2^ or 300/km^2^), and baiting duration in years. Year and study cell were treated as random effects. For sex, we coded females as 0, males as 1 and those missing data as 0.5. We used yearly age classes based on cementum annuli counts when available. If an animal was recaptured in a different trapping period and missing cementum age data, we used a previous known age to estimate that animal’s age class for that trapping period. For individuals that had only relative age classifying them as a juvenile (<1 year), age class was listed as zero. For adult raccoons and skunks with unknown age class, we coded the records as 2.35 years for raccoons and 1.46 years for skunks, which was the mean of the negative binomial distribution fit to the adult age data for each species. Competitive models were considered as having a change of ≤ 2 in AICc from the top model.

## 3. Results

Cumulatively over the five-year study period, 1,506,678 baits were distributed by either air or ground means (Table 2). During the period 2012–2013, when the target bait density was 75/km^2^, an average of 132,339 (±479 SD) baits were distributed/year. During the period 2014–2016, when the target bait density was 300/km^2^, an average of 414,000 (±4124 SD) baits were distributed/year. We processed sera for RVNA from 3256 carnivores sampled during the period 2012–2016. Raccoons accounted for 90.8% (*n* = 2955) of animals captured, followed by striped skunks (8.9%; *n* = 290). Red foxes, gray foxes, and coyotes represented less than 1.0% of captures. Raccoons and skunks were recaptured during 619 and 50 events, respectively, and all events were included in total count of captures per species. During this study, 20 target animals were euthanized, one was found dead (i.e., roadkill), and four died under care; 64% (16/25) of the animals were tested for rabies. Two striped skunks tested positive with the raccoon RV variant during 2013, whereas 14 other animals tested negative for rabies during the period 2012–2015. The two positive skunks were euthanized for abnormal behavior and the presence of a lesion. During 2016, no study animals were submitted for testing.

**Figure 2:**
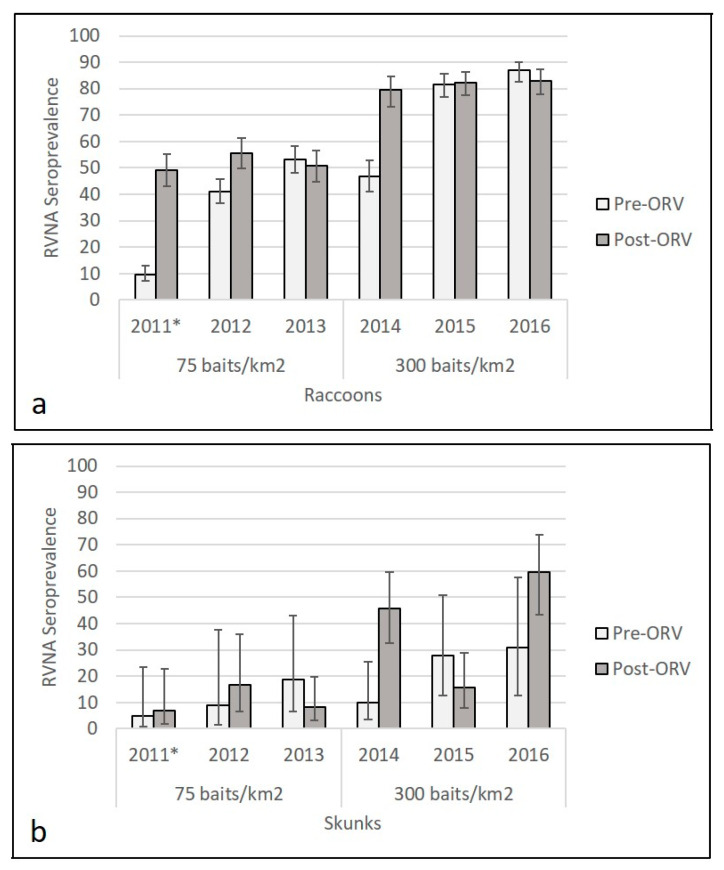
Raccoon (**a**) and skunk (**b**) rabies virus-neutralizing antibody (RVNA) seroprevalence from oral rabies vaccination (ORV) field trials with Ontario Rabies Vaccine Baits (ONRAB) in West Virginia, USA in relationship to bait density, sampling period (pre- or post-ORV), and year. RVNA cutoff observed was 0.125 IU/mL for the period 2012–2016. * Information from 2011 was from Slate et. al. [32] and included for comparison with years 2012–2016.

Higher RVNA seroprevalence was observed in raccoon and skunk populations sampled across the high-density (300/km^2^) ORV during the period 2014–2016 (Figure 2, Table S3). For reference in 2011, the area was naïve for ORV prior to the baiting that year; the published pre-ORV seroprevalence was 9.6% (38/395, 95% CI: 7.1–12.9%) among raccoons and 5% (1/20, 95% CI: 1–24%) among skunk populations and post-ORV seroprevalence that year was 49.2% (129/262, 95% CI 43.2–55.3) for raccoons and 7% (2/28, 95% CI 2–23%) for skunks [32]. Average pre-ORV seroprevalence in the study area at the 0.125 IU/mL cutoff for the two years (2012–2013) at 75 baits/km^2^ was 46.6% (362/776, 95% CI: 43.2–50.2%) for raccoon and 15% (4/27, 95% CI: 6–32%) for skunk populations. Average post-ORV seroprevalence during standard density (75 baits/km^2^) ORV during the period 2012–2013 was 53.1% (300/565, 95% CI: 50.0–57.2%) among raccoon and 11% (8/72, 95% CI: 6–20%) among skunk populations. In 2014, baiting transitioned to 300 baits/km^2^; pre-ORV seroprevalence was 46.9% (130/277, 95% CI 41.1–52.8) for raccoon and 10% (3/30, 95% CI 4–26%) for skunk populations and post-ORV seroprevalence that year was 79.5% (147/185, 95% CI 73.1–84.7) for raccoons and 46% (22/48, 95% CI 33–60%) for skunks. Average pre-ORV seroprevalence for the two years (2015–2016) following the initial 300 baits/km^2^ distribution in 2014 was 84.4% (514/609, 95% CI: 81.3–87.1%) for raccoon and 29% (9/31, 95% CI: 16–47%) for skunk populations. Average post-ORV seroprevalence at the high-density ORV during the period 2015–2016 was 82.7% (449/543, 95% CI: 79.3–85.6%) for raccoon and 35% (29/82, 95% CI: 26–48%) for skunk populations. At the higher cutoff of 0.5 IU/mL, we re-examined seroprevalence estimates for raccoon and skunk samples and continued to observe a higher seroprevalence for both raccoons and skunks at the 300 baits/km^2^ compared to 75 baits/km^2^ treatment (Figure S1, Table S4). A higher proportion of raccoons and skunks were marked with TTCC at 300 baits/km^2^ than at 75 baits/km^2^ (Figure S2).

The population-level GLMM indicated positive relationships between bait density and levels of RVNA among raccoon and skunk populations at the 0.125 IU/mL cutoff (Table 3). Among raccoon populations, the estimated seroprevalence increased post-ORV from 53% (95% CI: 49–56%) using 75 baits/km^2^ to 83% (95% CI: 81–85%) using 300 baits/km^2^. Seroprevalence pre-ORV increased over time for both bait densities, whereas there was no apparent trend for increasing seroprevalence post-ORV within the period 2012–2013 or the period 2014–2016, respectively (Figure S3). Among skunk populations, the estimated seroprevalence increased post-ORV from 16% (95% CI: 15–16%) at 75 baits/km^2^ to 38% (95% CI: 36–39%) at 300 baits/km^2^. The duration of baiting increased seroprevalence pre-ORV for both bait densities, but this trend was not observed post-ORV (Figure S3). Similar trends for the GLMM were observed among raccoon population pre- and post- ORV and among skunk populations pre-ORV when modeling the seroprevalence response data at the 0.5 IU/mL cutoff (Table S5, Figure S3). Model selection supported bait density as a key factor influencing raccoon and skunk population seroprevalence (Supplementary File and Tables S11–S15).

**Figure 3:**
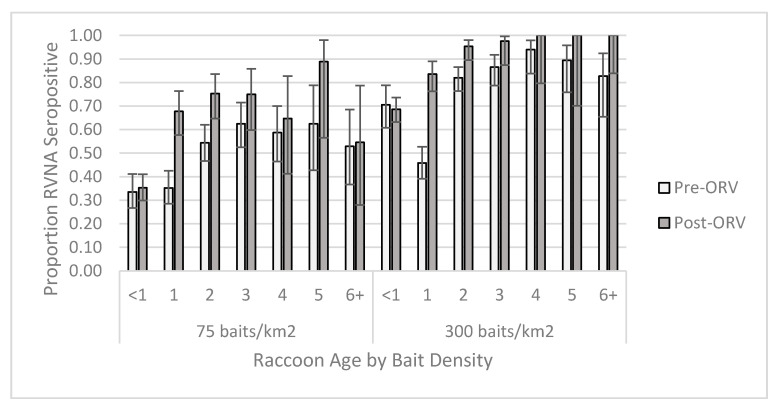
Proportion of raccoons seropositive for rabies virus-neutralizing antibodies (RVNA) at a cutoff of 0.125 IU/mL by year of age and bait densities sampled pre- and post-oral rabies vaccination (ORV) in West Virginia, USA. Error bars represent 95% confidence intervals.

**Figure 4:**
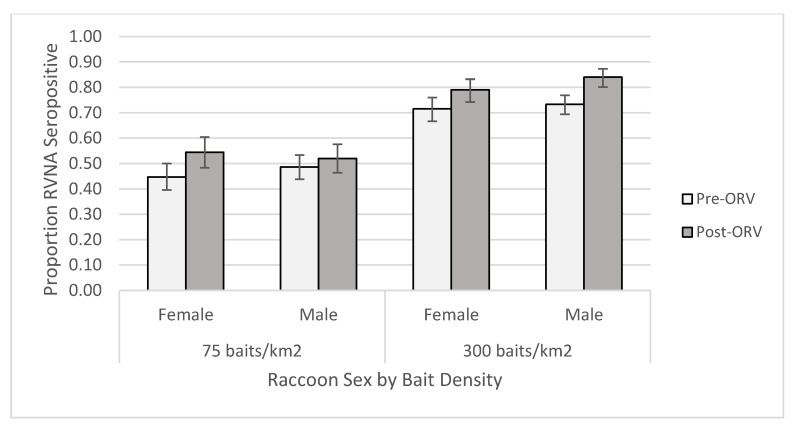
Proportion of raccoons seropositive for rabies virus-neutralizing antibodies (RVNA) at a cutoff of 0.125 IU/mL by sex and bait densities sampled pre- and post-oral rabies vaccination (ORV) in West Virginia, USA. Error bars represent 95% confidence intervals.

For the individual-level GAM at the 0.125 IU/mL cutoff, the raccoon dataset yielded one competitive model (Table S6) and skunk dataset had six competitive models (Table S7). The raccoon top model contained two-way interactions of period *x* age and bait density *x* years of baiting, in addition to the main effect terms and sex (Table S8). This model had a marginalized and conditional R^2^ of 0.270. Older raccoons were more likely to be seropositive, especially during the post-ORV period (Figure 3). Raccoons captured during ORV using 300 baits/km^2^ were also more likely to be seropositive with multiple years of baiting also increasing the probability of being seropositive. Male raccoons were more likely to be seropositive than females regardless of a slightly higher proportion of females positive at the 75 baits/km^2^ post-ORV (Figure 4). The top skunk model contained the main effects of period, bait density, and years of baiting (Table S9) and had a marginalized and conditional R^2^ of 0.136. Skunks captured during 300 baits/km^2^ ORV, during a post-ORV period, or following more years of baiting were more likely to be seropositive. Age (Figure S4) and sex (Figure S5) were not in the top skunk model, but sex was in a competitive model (Table S9). All six of the competing models contained the main effects of bait density and period.

## 4. Discussion

The standard ORV bait density used to target raccoons in the USA is 75 baits/km^2^. However, it has been recognized since the early years of ORV in the USA that there was no compelling scientific basis for a constant bait density of 75 baits/km^2^ across all raccoon populations in the eastern USA and the NRMP has reported use of 150 baits/km^2^ to target raccoon populations in urban or higher risk areas (e.g., Selma, AL [44]; St. Lawrence River, NY [45]; Pinellas County, FL [46]; Vermont-Quebec border [27]). Early testing regarding the population-level response to ORV using target bait densities of 75, 150, and 300/km^2^ in Ohio showed higher seroprevalence among raccoons as RABORAL V-RG bait density increased [47]. In spite of this positive early response to ORV in Ohio, the magnitude of the population seroprevalence response did not meet the theoretical vaccination coverage (≥60%) recommended for effective raccoon RV control and elimination [48,49]. Pedersen et al. [50] reported a similar trend for RABORAL V-RG in a Pennsylvania field trial with target bait densities approximating those evaluated by Sattler et al. [47], yet a subsequent study at 75/km^2^ and 150/km^2^ in Virginia indicated no change in seroprevalence between treated target populations [51].

The initial 2011 ONRAB field trial in the USA resulted in a 49.2% raccoon seroprevalence post-ORV, the highest observed for an initial baiting at 75 baits/km^2^ in an ORV-naïve, raccoon RV enzootic area [32]. The post-ORV seroprevalence during the period 2012–2013 remained similar for raccoons at 53.1%. While raccoon cases were in decline by 2013 in the study area, skunk cases were still being detected during 2013 and the field trial was continued during the period 2014–2016, but with a greater target bait density of 300/km^2^ in view of the encouraging response observed by Rosatte et al. [5] targeting control of arctic fox RV in skunks in southern Ontario. Our high-density ORV did result in higher RVNA seroprevalence in raccoons and skunks, regardless of IU/mL cutoff level applied. The highest level of seroprevalence we observed post-ORV was 83.1% for raccoons and 60% for skunks and both occurred during the final year of this field trial (Figure 2).

Our results can be compared to the ONRAB field evaluation that occurred at 75 baits/km^2^ in the northeastern USA (rural northeastern NY and northern VT and NH) [28] and in the St. Lawrence region of NY [29]. For raccoon populations baited annually at 75 baits/km^2^ and evaluated for RVNA seroprevalence using the same serologic methods, West Virginia and the St. Lawrence region had comparable average post-seroprevalence, which was 53.1% (95% CI 50.0–57.2%) and 55.9% (95% CI 53.1–60.8%) [29], respectively, while the post-ORV seroprevalence was 68.5% (95% CI 66.2–70.8%) for the northeastern USA field trial site [28], commensurate with enhanced progress in regional raccoon RV elimination [20]. Any factor that influences bait uptake and seroprevalence may have contributed to differences among locations. Possible factors that may negatively influence bait uptake are increased non-target bait competition, higher raccoon densities, smaller home ranges, and higher availability of anthropogenic food resources [52,53,54].

Age was a factor related to seroprevalence in West Virginia, the St. Lawrence region and northeastern USA, as well as southern Quebec, Canada [55]. This consistent result across studies and regions suggests that the level of seroprevalence estimated can depend on both the recruitment rate of juveniles into the adult population and survival of previously vaccinated animals. We observed a saturating effect in RVNA seroconversion among raccoons for both bait density applications and following successive years of ORV, which had been an expectation from the early report regarding the first year of evaluation in West Virginia [32]. Other studies have observed a similar trend with targeted raccoon populations [28,47,55]. Even when seroprevalence may increase with years of baiting, population turnover can diminish seroprevalence gains within a few years once management activities cease. Over 85% of the raccoons sampled were ≤3 years of age in this study (Figure S6) and in Gilbert et al. [28]; both observed a serologic asymptote after two years of baiting. Thus for raccoons, continued baiting past two or three years without modifying the baiting strategy may be unlikely to result in further increases in RVNA seroprevalence.

For skunks captured during ONRAB field trials in the USA, the average post-ORV RVNA response at 75 baits/km^2^ was similar among the different regions at 18% (95% CI 13–26%), 19% (95% CI 13–27%), and 11% (95% CI 6–20%) for the northeastern USA, St. Lawrence region, and West Virginia, respectively. Also observed among these studies was the occasional year where the measured pre-ORV seroprevalence among skunks appeared higher than post-ORV seroprevalence in that same year (e.g., 2014 in northeastern USA, 2014 in St. Lawrence region, and 2013 in West Virginia). In all of these years, the actual number of seropositive skunks post-ORV was the same or higher than the number seropositive skunks pre-ORV, but the total captures post-ORV was greater than the pre-ORV period indicating that at least some of the variability in seroprevalence estimates is likely influenced by overall and seasonal capture success. Given that raccoons were the focal target of these ORV field trials and their evaluation, factors such as sex and age that may have influenced skunk seroprevalence were not examined and thus, were not available for comparison. In southern Quebec, skunk age, sex, weighted bait density, habitat type, and number of ONRAB ORV campaigns at 43–155 baits/km^2^ were evaluated, but none of these variables explained the variation observed in skunk population seropositivity [55]. We found that skunks sampled at 300 baits/km^2^, post-ORV, or in later years of baiting were more likely to be seropositive at the lower RVNA threshold cutoff employed. We also observed that older age, greater bait density and increased years of baiting were associated with RVNA seroconversion at the higher RVNA threshold cutoff (of 0.5 IU/mL). We examined two different assay cutoffs since the lower RVNA is relevant to raccoon rabies management in the USA [56] and the higher cutoff is relevant for comparisons with wildlife rabies management in other countries (e.g., Canada). The Supplementary File, Figures S1 and S3, Tables S4, S5, S11, S13 and S15–S19 provides additional results and discussion at the 0.5 IU/mL cutoff. Given that 99% of the skunks we sampled were ≤3 years of age (Figure S7), we might expect a more rapid population turnover cycle with striped skunks compared to raccoons.

Estimates of RVNA seroprevalence and rabies case reduction are two key metrics used by the NRMP for monitoring ORV and related activities (e.g., trap-vaccinate-release). RVNA seroprevalence is a correlate of survival among orally vaccinated target wildlife against lethal rabies virus infection [57] even when the presence of the some RVNA (e.g., detection in a pre-ORV period in a ORV-naïve area) may be due to natural sublethal RV exposures [8,58,59]. Just as there is no specific ORV bait density to uniformly apply to all target populations, there is not one level of seroprevalence needed to eliminate raccoon RV in all places. Theoretical estimates of population immunity for elimination range from 50% to 100% for foxes [60,61,62], are similar for raccoons [48,63,64], and are underdeveloped for skunk populations. This variation in the vaccination coverage necessary for elimination may be due to population density, movement patterns, contact rates and enzootic versus epizootic phases of transmission [48,65,66,67] as well as other potential factors such as the size and scale of landscapes and populations under consideration. RVNA seroprevalence measured for the raccoon population in WV at both bait densities (53% at 75 baits/km^2^ and 82% at 300 baits/km^2^) is within the theoretical estimates for rabies control and elimination.

The other key metric for evaluating ORV success is case reduction confirmed through enhanced rabies surveillance [68]. Occasionally, a natural reduction in incidence can occur without management intervention or management intervention may not be the only cause for case reduction [69,70]. Our study area prior to 2011 was enzootic for raccoon RV with detection of ≥10 raccoon RV cases annually (Table 4). Cases in raccoons were no longer detected after 2011, but raccoon RV continued to be detected from skunks during the period 2012–2014. After one year of ONRAB distribution at 300 baits/km^2^, no further cases of raccoon RV were detected. Furthermore, raccoon RV cases in any species have not been detected in years following this study (through 2019). The timing of the ORV management interventions used in this study are associated with locally observed case reductions.

## 5. Conclusions

Increasing ONRAB bait density from 75/km^2^ to 300/km^2^ resulted in robust RVNA seroprevalence among raccoons and skunks sampled. Rabies cases in the study area declined to zero across multiple years of application but the risk for re-infection with raccoon RV could return after a few years post-management and depends on spatial landscape context and the ecology and connectivity of reservoir populations. The higher-density baiting treatment is prohibitively costly over large landscapes for the control and prevention of raccoon RV, but may be strategically employed for elimination in high-risk areas including areas with elevated risk of spillover to wildlife and domesticated animals.

## Figures and Tables

**Table 1:** Trapping dates for pre- and post-oral rabies vaccination (ORV) by year and Ontario Rabies Vaccine Bait (ONRAB) density in West Virginia, USA.

Year	Period	Trapping Dates	Bait Density (Baits/km^2^)
2012	Pre-ORV	17 July–10 August 2012	75
	Post-ORV	3–13 October 2012	75
2013	Pre-ORV	16 July–9 August 2013	75
	Post-ORV	22 October–1 November 2013	75
2014	Pre-ORV	17 July–3 August 2014	300
	Post-ORV	6–19 October 2014	300
2015	Pre-ORV	21 July–1 August 2015	300
	Post-ORV	5–17 October 2015	300
2016	Pre-ORV	19–29 July 2016	300
	Post-ORV	11–21 October 2016	300

**Table 2:** Number of Ontario Rabies Vaccine Baits (ONRAB) distributed and overall area covered during oral rabies vaccination by aerial or ground distribution methods at target bait densities of 75/km^2^ in the period 2012–2013 and 300 /km^2^ in the period 2014–2016 in West Virginia, USA.

	Aerial		Aerial Dates	Ground		Ground Dates	Total Baits	Total Area (km^2^)	Targeted Bait Density
Year	Baits	Area (km^2^)		Baits	Area (km^2^)				(Baits/km^2^)
2012	131,154	2343	26 August 2012	1524	27	28 August 2012	132,678	2370	75
2013	130,476	2300	3 September 2013	1524	27	27 August 2013	132,000	2327	75
2014	412,200	1864	26–28 August 2014	6300	27	27 August 2014	418,500	1891	300
2015	404,100	1864	26–27 August 2015	6300	27	27 August 2015	410,400	1891	300
2016	406,800	1864	30 August–2 September 2016	6300	27	31 August 2016	413,100	1891	300

**Table 3:** Covariate estimates for generalized linear mixed model on raccoon and skunk seroprevalence from oral rabies vaccination (ORV) at the 0.125 IU/mL cutoff in West Virginia, USA.

Species	Parameter	Estimate	Std. Error	z Value	Pr (>|z|)
Raccoons	Intercept	−2.75	0.40	−6.81	0
	Bait density	0.01	0.00	9.10	0
	Years of baiting	0.88	0.15	6.01	0
	Period (pre- or post-ORV)	2.23	0.49	4.58	0
	Years of baiting X period	−0.83	0.21	−4.01	0
Skunks	Intercept	−3.96	0.83	−4.79	0.00
	Bait density	0.01	0.00	3.71	0.00
	Years of baiting	0.66	0.28	2.35	0.02
	Period (pre- or post-ORV)	1.71	0.91	1.88	0.06
	Years of baiting X period	−0.60	0.38	−1.56	0.12

**Table 4:** Raccoon rabies virus variant surveillance from 2008 to 2019 in the area of the Ontario Rabies Vaccine Bait (ONRAB) trials in West Virginia, USA. Data are a combination of direct fluorescent antibody testing performed by the West Virginia Department of Health and Human Resources (WVDHHR; data used with permission) and direct rapid immunohistochemistry testing performed by the United States Department of Agriculture, Wildlife Services.

Year	Negatives	Positives	Rabid Raccoons	Rabid Skunks	Rabid Others ^1^
2008	33	10	3	5	2
2009	75	33	23	8	2
2010	113	31	13	15	3
2011	81	34	14	19	1
2012	88	3	0	3	0
2013	47	9	0	9	0
2014	54	2	0	2	0
2015	59	0	0	0	0
2016	32	0	0	0	0
2017	30	0	0	0	0
2018 ^2^	19	0	0	0	0
2019	56	0	0	0	0

^1^ Other rabid animals with the raccoon rabies virus variant included one fox and one goat in 2018; one sheep and one woodchuck in 2009; one dog, one fox and one gray fox in 2010; and one cat in 2011. ^2^ Missing WVDHHR-negative data.

## Data Availability

The data presented in this study are available in this article and its Supplemental Material.

## Supplementary Materials

The following are available online at https://www.mdpi.com/1999-4915/13/2/157/s1, Files: Expanded results on (1) tetracycline (TTCC) biomarker observed, (2) generalized liner mixed modeling (GLMM) for rabies virus-neutralizing antibodies (RVNA) at 0.5 IU/mL cutoff, for TTCC, and for model selection, and (3) generalized additive modeling (GAM) for RVNA at 0.5 IU/mL cutoff and TTCC biomarker. Figure S1: Raccoon (a) and skunk (b) rabies virus-neutralizing antibody (RVNA) seroprevalence from oral rabies vaccination (ORV) field trials with Ontario Rabies Vaccine Baits (ONRAB) in West Virginia, USA in relationship to bait density, sampling period (pre- or post-ORV), year. RVNA cutoff observed was 0.5 IU/mL). Error bars reflect the 95% confidence interval. Figure S2: Proportion of (a) raccoons and (b) skunks positive for tetracycline hydrochloride (TTCC) biomarker from the oral rabies vaccination (ORV) field trials with Ontario Rabies Vaccine Baits (ONRAB) in West Virginia, USA in relationship to bait density, sampling period (pre- or post-ORV), and year. A total of 2,329 samples from raccoons and 204 samples from skunks were evaluated. Error bars reflect the 95% confidence interval. Figure S3: Estimated raccoon and skunk rabies virus-neutralizing antibody (RVNA) seroprevalence from oral rabies vaccination (ORV) field trials with Ontario Rabies Vaccine Baits (ONRAB) in West Virginia, USA in relationship to bait density, sampling period (pre- or post-ORV), and year. Estimates were from a beta regression analysis using RVNA cutoffs at 0.125 IU/mL (a: raccoons and b: skunks) and at 0.5 IU/mL (c: raccoons and d: skunks). Shading represents the 95% confidence interval. Figure S4: Proportion of skunks seropositive for rabies virus-neutralizing antibodies (RVNA) at a cutoff of 0.125 IU/mL by age and bait densities sampled pre- and post-oral rabies vaccination (ORV) in West Virginia, USA. Error bars represent 95% confidence intervals. Figure S5: Proportion of skunks seropositive for rabies virus-neutralizing antibodies (RVNA) at a cutoff of 0.125 IU/mL by sex and bait densities sampled pre- and post-oral rabies vaccination (ORV) in West Virginia, USA. Error bars represent 95% confidence intervals. Figure S6: Histogram of raccoon (n = 2296) ages based on cementum annuli of premolar teeth from the oral rabies vaccination (ORV) field trials with Ontario Rabies Vaccine Baits (ONRAB) in West Virginia, USA. The oldest raccoon was 15 years old. Figure S7: Histogram of skunk (n = 192) ages based on cementum annuli of premolar teeth from the oral rabies vaccination (ORV) field trials with Ontario Rabies Vaccine Baits (ONRAB) in West Virginia, USA. The oldest skunk was 4 years old. Figure S8: Estimated prevalence of the biomarker tetracycline hydrochloride (TTCC) in raccoon (a) and skunk (b) populations from oral rabies vaccination (ORV) field trials with Ontario Rabies Vaccine Baits (ONRAB) in West Virginia, USA in relationship to bait density, sampling period (pre- or post-ORV), and year. Estimates were from a beta regression analysis and shading represents the 95% confidence interval. Table S1: Comparison of rabies virus-neutralizing antibody (RVNA) results for a subset of raccoon and skunk serum samples tested at New York State Department of Health (NYSDOH; lab 1) and Kansas State University (lab 2). The RVNA titers were determined from all raccoon and skunk samples at NYSDOH using a cutoff at 0.125 IU/mL. A subset of 300 raccoon samples and 39 skunk samples were tested at lab 2, which used a cutoff of 0.1 IU/mL. Table S2: The models compared for the individual-level generalized additive modeling analysis for the raccoons and skunks. The same models were used for both species and with three different response variables; which were (1) rabies virus-neutralizing antibodies (RVNA) with a seropositive cutoff at 0.125 IU/mL, (2) RVNA at 0.5 IU/mL cutoff, and (3) prevalence of the tetracycline biomarker Within the structure of the models, “*” indicates both an additive and interaction of the variables surrounding the “*”. Table S3: Information on target species sampling (n), rabies virus-neutralizing antibody (RVNA) seroprevalence (%) at 0.125 IU/mL cutoff and 95% confidence interval for oral rabies vaccination with Ontario Rabies Vaccine Baits (ONRAB) at 75 baits/km^2^ in the period 2012–2013 and at 300 baits/km^2^ in the period 2014–2016 in West Virginia, USA. Due to sequential baiting in the cells for the different bait densities, the year 2014 was split; the pre-ORV trapping period had previously been baited at 75 baits/km^2^ and it was only the post-ORV trapping period that followed baiting at 300 baits/km^2^. Table S4. Information on target species sampling (n), rabies virus-neutralizing antibody (RVNA) seroprevalence (%) at the 0.5 IU/mL cutoff and 95% confidence interval for oral rabies vaccination with Ontario Rabies Vaccine Baits (ONRAB) at 75 baits/km^2^ in the period 2012–2013 and at 300 baits/km^2^ in the period 2014–2016 in West Virginia, USA. Due to sequential baiting in the cells for the different bait densities, the year 2014 was split; the pre-ORV trapping period had previously been baited at 75 baits/km^2^ and it was only the post-ORV trapping period that followed baiting at 300 baits/km^2^. All sampled coyotes (n = 1) and foxes (n = 10) were negative at 0.5 IU/mL and are not included in this table. Table S5: Covariate estimates for generalized linear mixed model on raccoon and skunk seroprevalence from oral rabies vaccination (ORV) at the 0.5 IU/mL cutoff in West Virginia, USA. Table S6: Model selection table of the individual-level generalized additive modeling candidate model set for raccoon seroprevalence at the 0.125 IU/mL cutoff. Models are sorted from most parsimonious on top to least parsimonious based on Akaike’s information criterion corrected (AICc) for small sample sizes. The model names, degrees of freedom (df), the log likelihood (LL), the AICc, the delta AICc (difference between the model AICc value and the top model), the model likelihood(ModelLik), the AICc weight (relative support for each model), and the cumulative AICc weight (Cum.Wt, representing total cumulative weight for the given model and all models above it) are shown. Table S7: Model selection table of the individual-level generalized additive modeling candidate model set for skunk seroprevalence at the 0.125 IU/mL cutoff. Models are sorted from most parsimonious on top to least parsimonious based on Akaike’s information criterion corrected (AICc) for small sample sizes. The model names, degrees of freedom (df), the log likelihood (LL), the AICc, the delta AICc (difference between the model AICc value and the top model), the model likelihood(ModelLik), the AICc weight (relative support for each model), and the cumulative AICc weight (Cum.Wt, representing total cumulative weight for the given model and all models above it) are shown. Table S8: Parameters and estimates of the top competitive models for the individual-level generalized additive modeling for raccoons based on a rabies virus-neutralizing antibody (RVNA) cutoff of 0.125 IU/mL. Oral rabies vaccination (ORV) occurred with Ontario Rabies Vaccine Baits (ONRAB) in West Virginia, USA. Table S9: Parameters and estimates of the top competitive models for the individual-level generalized additive modeling for skunks based on a rabies virus-neutralizing antibody (RVNA) cutoff of 0.125 IU/mL. Oral rabies vaccination (ORV) occurred with Ontario Rabies Vaccine Baits (ONRAB) in West Virginia, USA. Table S10: Covariate estimates for generalized linear mixed model on portion of the tetracycline marked raccoons and skunks from oral rabies vaccination (ORV) in West Virginia, USA. Table S11: The models compared for the generalized liner mixed model analysis for the raccoons and skunks. The same models were used for both species and with two different response variables; which were (1) rabies virus-neutralizing antibodies (RVNA) with a seropositive cutoff at 0.125 IU/mL and (2) RVNA at 0.5 IU/mL cutoff. Within the structure of the models, “*” indicates both an additive and interaction of the variables surrounding the “*”. Table S12: Model selection table of the generalized liner mixed candidate model set for raccoon seroprevalence at the 0.125 IU/mL cutoff. Models are sorted from most parsimonious on top to least parsimonious based on Akaike’s information criterion corrected (AICc) for small sample sizes. The model names, number of parameters (*k*), the AICc, the delta AICc (difference between the model AICc value and the top model), the model likelihood(ModelLik), the AICc weight (relative support for each model), the log likelihood (LL), and the cumulative AICc weight (Cum.Wt, representing total cumulative weight for the given model and all models above it) are shown. Table S13: Model selection table of the generalized liner mixed candidate model set for raccoon seroprevalence at the 0.5 IU/mL cutoff. Models are sorted from most parsimonious on top to least parsimonious based on Akaike’s information criterion corrected (AICc) for small sample sizes. The model names, number of parameters (*k*), the AICc, the delta AICc (difference between the model AICc value and the top model), the model likelihood(ModelLik), the AICc weight (relative support for each model), the log likelihood (LL), and the cumulative AICc weight (Cum.Wt, representing total cumulative weight for the given model and all models above it) are shown. Table S14: Model selection table of the generalized liner mixed candidate model set for skunk seroprevalence at the 0.125 IU/mL cutoff. Models are sorted from most parsimonious on top to least parsimonious based on Akaike’s information criterion corrected (AICc) for small sample sizes. The model names, number of parameters (*k*), the AICc, the delta AICc (difference between the model AICc value and the top model), the model likelihood(ModelLik), the AICc weight (relative support for each model), the log likelihood (LL), and the cumulative AICc weight (Cum.Wt, representing total cumulative weight for the given model and all models above it) are shown. Table S15: Model selection table of the generalized liner mixed candidate model set for skunk seroprevalence at the 0.5 IU/mL cutoff. Models are sorted from most parsimonious on top to least parsimonious based on Akaike’s information criterion corrected (AICc) for small sample sizes. The model names, number of parameters (*k*), the AICc, the delta AICc (difference between the model AICc value and the top model), the model likelihood(ModelLik), the AICc weight (relative support for each model), the log likelihood (LL), and the cumulative AICc weight (Cum.Wt, representing total cumulative weight for the given model and all models above it) are shown. Table S16: Model selection table of the individual-level generalized additive modeling candidate model set for raccoon seroprevalence at the 0.5 IU/mL cutoff. Models are sorted from most parsimonious on top to least parsimonious based on Akaike’s information criterion corrected (AICc) for small sample sizes (AICc). The model names, degrees of freedom (df), the log likelihood (LL), the AICc, the delta AICc (difference between the model AICc value and the top model), the model likelihood(ModelLik), the AICc weight (relative support for each model), and the cumulative AICc weight (Cum.Wt, representing total cumulative weight for the given model and all models above it) are shown. Table S17: Model selection table of the individual-level generalized additive modeling candidate model set for skunk seroprevalence at the 0.5 IU/mL cutoff. Models are sorted from most parsimonious on top to least parsimonious based on Akaike’s information criterion corrected (AICc) for small sample sizes. The model names, degrees of freedom (df), the log likelihood (LL), the AICc, the delta AICc (difference between the model AICc value and the top model), the model likelihood(ModelLik), the AICc weight (relative support for each model), and the cumulative AICc weight (Cum.Wt, representing total cumulative weight for the given model and all models above it) are shown. Table S18: Parameters and estimates of the top competitive models for the individual-level generalized additive modeling for raccoons based on a rabies virus-neutralizing antibody (RVNA) cutoff at 0.5 IU/mL. Oral rabies vaccination (ORV) occurred with Ontario Rabies Vaccine Baits (ONRAB) in West Virginia, USA. Table S19: Parameters and estimates of the top competitive models for the individual-level generalized additive modeling for skunks based on a rabies virus-neutralizing antibody (RVNA) cutoff at 0.5 IU/mL. Oral rabies vaccination (ORV) occurred with Ontario Rabies Vaccine Baits (ONRAB) in West Virginia, USA. Table S20: Model selection table of the individual-level generalized additive modeling candidate model set for raccoons based on detection of the tetracycline biomarker. Models are sorted from most parsimonious on top to least parsimonious based on Akaike’s information criterion (AICc) corrected for small sample sizes. The model names, degrees of freedom (df), the log likelihood (LL), the AICc, the delta AICc (difference between the model AICc value and the top model), the model likelihood(ModelLik), the AICc weight (relative support for each model), and the cumulative AICc weight (Cum.Wt, representing total cumulative weight for the given model and all models above it) are shown. Table S21: Model selection table of the individual-level generalized additive modeling candidate model set for skunks based on detection of the tetracycline biomarker. Models are sorted from most parsimonious on top to least parsimonious based on Akaike’s information criterion corrected (AICc) for small sample sizes. The model names, degrees of freedom (df), the log likelihood (LL), the AICc, the delta AICc (difference between the model AICc value and the top model), the model likelihood(ModelLik), the AICc weight (relative support for each model), and the cumulative AICc weight (Cum.Wt, representing total cumulative weight for the given model and all models above it) are shown. Table S22: Parameters and estimates of the top competitive models for the individual-level generalized additive modeling for raccoons based on detection of the tetracycline biomarker. Oral rabies vaccination (ORV) occurred with Ontario Rabies Vaccine Baits (ONRAB) in West Virginia, USA. Table S23: Parameters and estimates of the top competitive models for the individual level generalized additive modeling for skunks based on detection of the tetracycline biomarker. Oral rabies vaccination (ORV) occurred with Ontario Rabies Vaccine Baits (ONRAB) in West Virginia, USA.
