# Supplementary material for: Serological Responses of Raccoons and Striped Skunks to Ontario Rabies Vaccine Bait in West Virginia during 2012–2016
Source: Viruses. 2021 Jan 22;13(2):157. doi: 10.3390/v13020157 (PMC7912576; doi:10.3390/v13020157)
Supplement: Supplementary file 1 [file viruses-13-00157-s001.pdf]

Supplemental Files

Expanded results on 1) tetracycline (TTCC) biomarker observed, 2) generalized liner mixed modeling (GLMM) for rabies virus neutralizing antibodies (RVNA) at 0.5 IU/mL cutoff, for TTCC, and for model selection, and 3) generalized additive modeling (GAM) for RVNA at 0.5 IU/mL cutoff and TTCC biomarker.

Results

Tetracycline (TTCC) biomarker observed

Average pre-ORV prevalence of TTCC marked animals in the study area with a prior ONRAB standard density ORV (75 baits/km^2; years 2012-2014) was 30.3% (248/818 95% CI: 27.3-33.6%) for raccoon and 0.0% (0/33, 95% CI: 0-10.4%) for skunk populations. Average post-ORV prevalence of TTCC marked animals during standard density (75 baits/km^2) ORV during 2012-2013 was 40.7% (201/494, 95% CI: 36.5-45.1%) among raccoon and 0.2% (1/52, 95% CI: 0.0-10.1%) among skunk populations. Average pre-ORV prevalence of TTCC marked animals during high density ORV (300 baits/km^2; years 2015-2016) was 69.6% (311/447, 95% CI: 65.2-73.7%) for raccoon and 30.4% (7/23, 95% CI: 1.6-50.9%) for skunk populations. Average post-ORV prevalence of TTCC marked animals at the high density ORV was 74.9% (427/570, 95% CI: 71.2-78.3%) for raccoon and 24.0% (23/96, 95% CI: 1.7-33.4%) for skunk populations (Figure S1).

Generalized liner mixed modeling (GLMM)

RVNA seroprevalence 0.5 IU/mL cutoff

Modeling the response data at the 0.125 IU/mL and the 0.5 IU/mL cutoff resulted in similar trends for the GLMM, but the estimates in seroprevalence was lower at the 0.5 IU/mL for both the raccoon and skunk populations. The GLMM at the 0.5 IU/mL indicated positive relationships between bait density and levels of RVNA among raccoon and skunk populations (Table S5, Figure S3). Among raccoon populations, the estimated seroprevalence increased post-ORV from 39% (95% CI: 36-42%) using 75 baits/km^2 to 74% (95% CI: 71-77%) using 300 baits/km^2. Among skunk populations, the estimated seroprevalence increased post-ORV from 11% (95% CI: 10-11%) at 75 baits/km^2 to 27% (95% CI: 24-31%) at 300 baits/km^2.

TTCC response

Examination of TTCC in the raccoon and skunk populations indicated the same trend as the seroprevalence models, specifically that more animals were positively marked at 300 baits/km^2 than at 75 baits/km^2 (Figure S8). Among raccoon populations, the estimated marking prevalence increased post-ORV from 13% (95% CI: 11-14%) using 75 baits/km^2 to 53% (95% CI: 48-59%) using 300 baits/km^2. Among skunk populations, the estimated marking prevalence increased post-ORV from 9% (95% CI: 6-12%) at 75 baits/km^2 to 28% (95% CI: 15-42%) at 300 baits/km^2. Compared with the covariate estimates at the 0.125 IU/mL cutoff (Table 3) and the 0.5 IU/mL cutoff (Table S5), the TTCC bait density covariate estimate was higher for both raccoons and skunks (Table S10). Additionally for the skunks the graph indicate an increase in marked prevalence post-ORV as years of baiting increase, especially at the 300 baits/km^2.

Model selection

We compared nine models in our GLMM analysis to identify the factors influencing seroprevalence at both the 0.125 IU/mL and 0.5 IU/mL cutoff for both raccoons and skunks (Table S11). Only one competitive model existed for raccoons when the response variable was seroprevalence at 0.125 IU/mL cutoff (Table S12) and at the 0.5 IU/mL (Table S13) and its was the same model for both cut-offs. This model contained bait density and the two-way interaction years of baiting x period indicating that all of these factors compared were important to the seroprevalence. Four competitive models existed for skunks at 0.125 IU/mL (Table S14). The top model contained the individual variables bait density and years of bating indicating that of the factors compared, both variables were significantly impacting seroprevalence. One

competitive model existed for skunks at 0.5 IU/mL (Table S15), which contained bait density and period (Table S15).

Generalized additive modeling (GAM)

RVNA seroprevalence 0.5 IU/mL cutoff

At the 0.5 IU/mL cutoff, we observed three competitive models for raccoons (Table S16) and three for skunks (Table S17). The top raccoon model was the same as the 0.125IU/mL cutoff and contained sex and age as well as two two-way interactions: period x age and bait density x years for baiting (Table S18). They had the same relationship associated with whether an individual would likely be positive as the top model at the 0.125 IU/mL cutoff. The top skunk model contained the two-way interaction bait density x years for baiting and the main effects age and period (Table S19). When these effects could be compared, the relationship with period was the same as the top model at the 0.125 IU/ml cutoff; the other factors, the interaction and age, were not in that model. An individual skunk captured in the post-ORV period and associated with a bait density of 300 baits/km2 was more likely to be seropositive. Older skunks were also more likely to be seropositive.

TTCC biomarker

We compared 75 models in our GAM analysis to identify factors influencing individual-animal seroprevalence at both the 0.125 IU/mL and 0.5 IU/mL cutoff for both raccoons and skunks. We used the same 75 models to identify factors influence TTCC biomarking at the individual-animal level (Table S2). We observed one competitive model for raccoons (Table S20) and four competitive models for skunks (Table S21). The raccoon top model was the full model tested (Table S22). For the effects that could be directly compared in the top models, the interaction term bait density x years of baiting had the same association as the model evaluating seroprevalence at 0.125 IU/mL and at 0.5 IU/mL. The skunk top model contained one two-way interaction of period x age and included the variables bait density, and years of baiting (Table S23). Compared with the top model for seroprevalence at 0.125 IU/mL, the effects that could be compared, bait density and years of bating, had the same trends; a skunk was more likely to be positive at the 300 baits/km^2 and after more years of baiting.

Summary

Our additional evaluations continued to support higher seroprevalence at 300 baits/km^2 compared to 75 baits/km^2 for both raccoons and skunks. The analysis of the biomarker also supported trends we observed with the seroprevalence data.

Figures

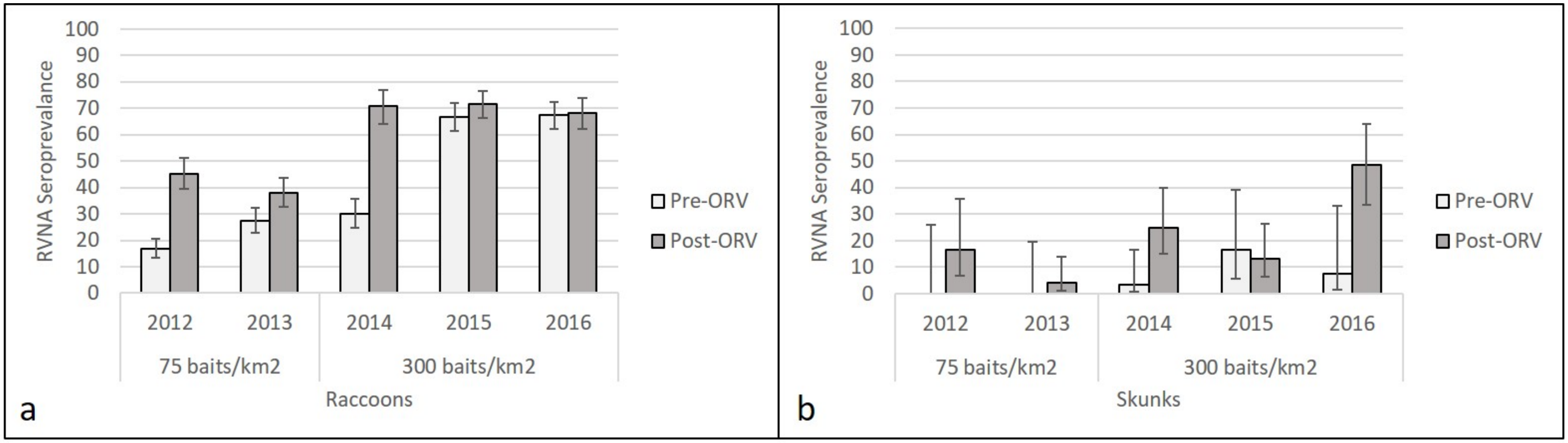

Figure S1: Raccoon (a) and skunk (b) rabies virus neutralizing antibody (RVNA) seroprevalence from oral rabies vaccination (ORV) field trials with Ontario Rabies Vaccine Baits (ONRAB) in West Virginia, USA in relationship to bait density, sampling period (pre- or post-ORV), year. RVNA cutoff observed was 0.5 IU/mL). Error bars reflect the 95% confidence interval.

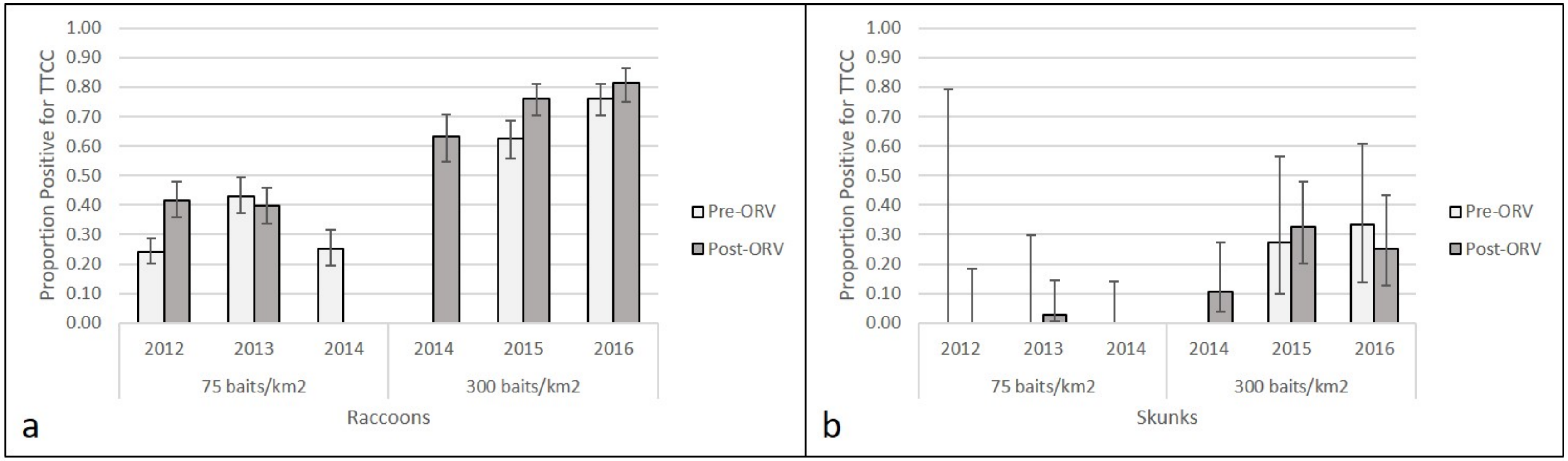

Figure S2: Proportion of (a) raccoons and (b) skunks positive for tetracycline hydrochloride (TTCC) biomarker from the oral rabies vaccination (ORV) field trials with Ontario Rabies Vaccine Baits (ONRAB) in West Virginia, USA in relationship to bait density, sampling period (pre- or post-ORV), and year. A total of 2,329 samples from raccoons and 204 samples from skunks were evaluated. Error bars reflect the 95% confidence interval.

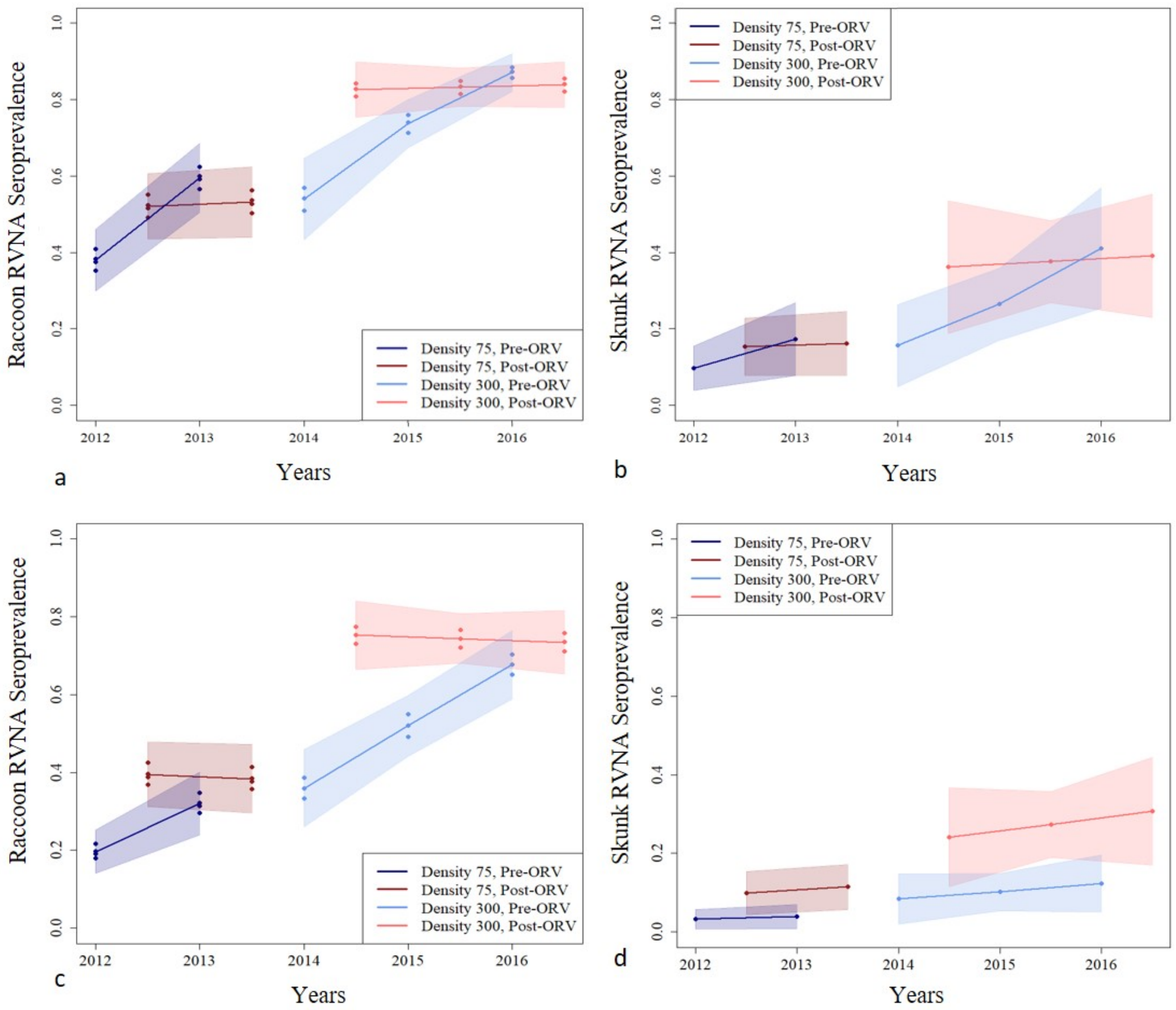

Figure S3: Estimated raccoon and skunk rabies virus neutralizing antibody (RVNA) seroprevalence from oral rabies vaccination (ORV) field trials with Ontario Rabies Vaccine Baits (ONRAB) in West Virginia, USA in relationship to bait density, sampling period (pre- or post-ORV), and year. Estimates were from a beta regression analysis using RVNA cutoffs at 0.125 IU/mL (a: raccoons and b: skunks) and at 0.5 IU/mL (c: raccoons and d: skunks). Shading represents the 95% confidence interval.

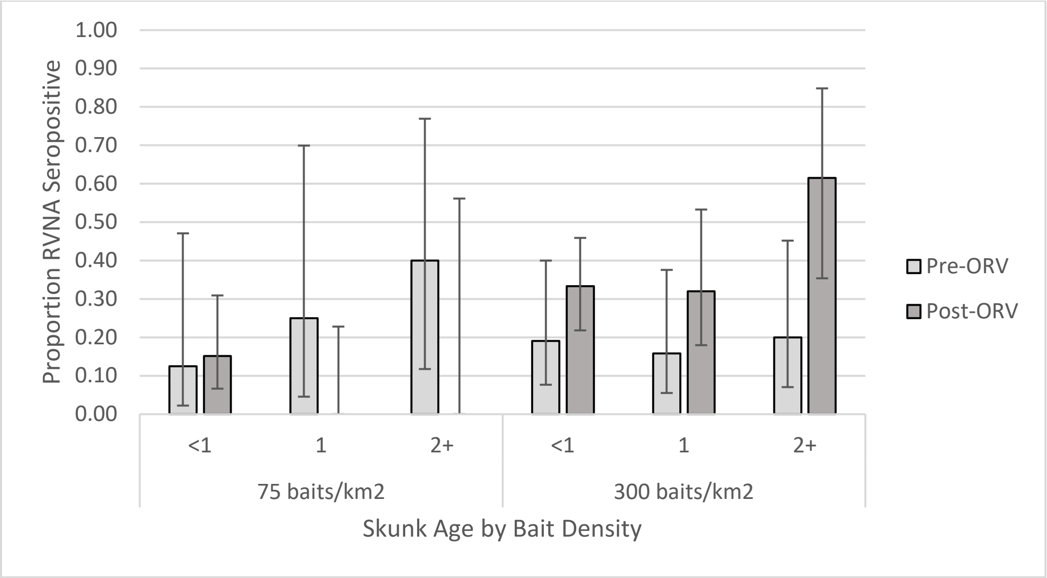

Figure S4: Proportion of skunks seropositive for rabies virus neutralizing antibodies (RVNA) at a cutoff of 0.125 IU/mL by age and bait densities sampled pre- and post-oral rabies vaccination (ORV) in West Virginia, USA. Error bars represent 95% confidence intervals.

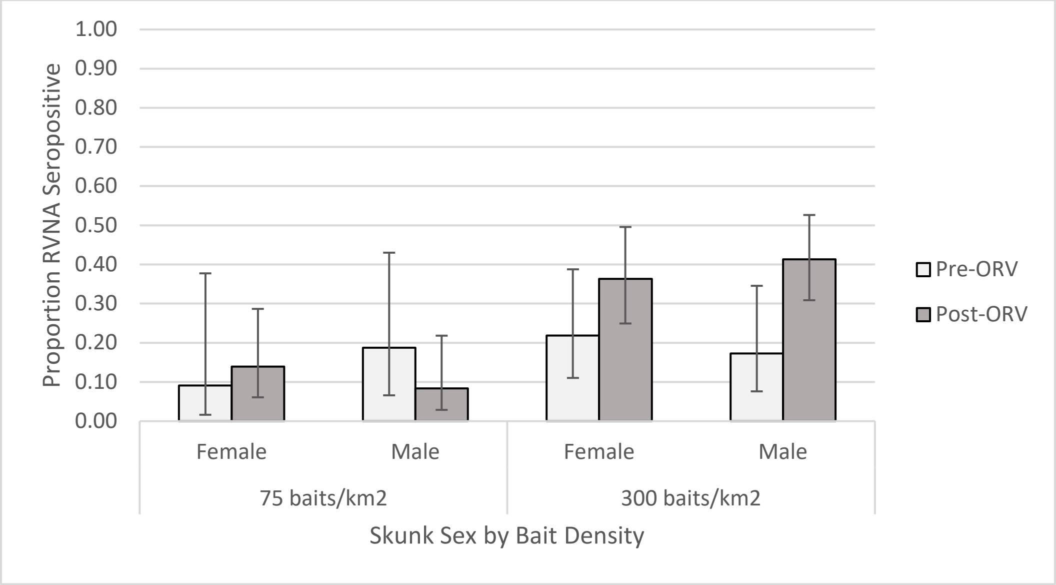

Figure S5: Proportion of skunks seropositive for rabies virus neutralizing antibodies (RVNA) at a cutoff of 0.125 IU/mL by sex and bait densities sampled pre- and post-oral rabies vaccination (ORV) in West Virginia, USA. Error bars represent 95% confidence intervals.

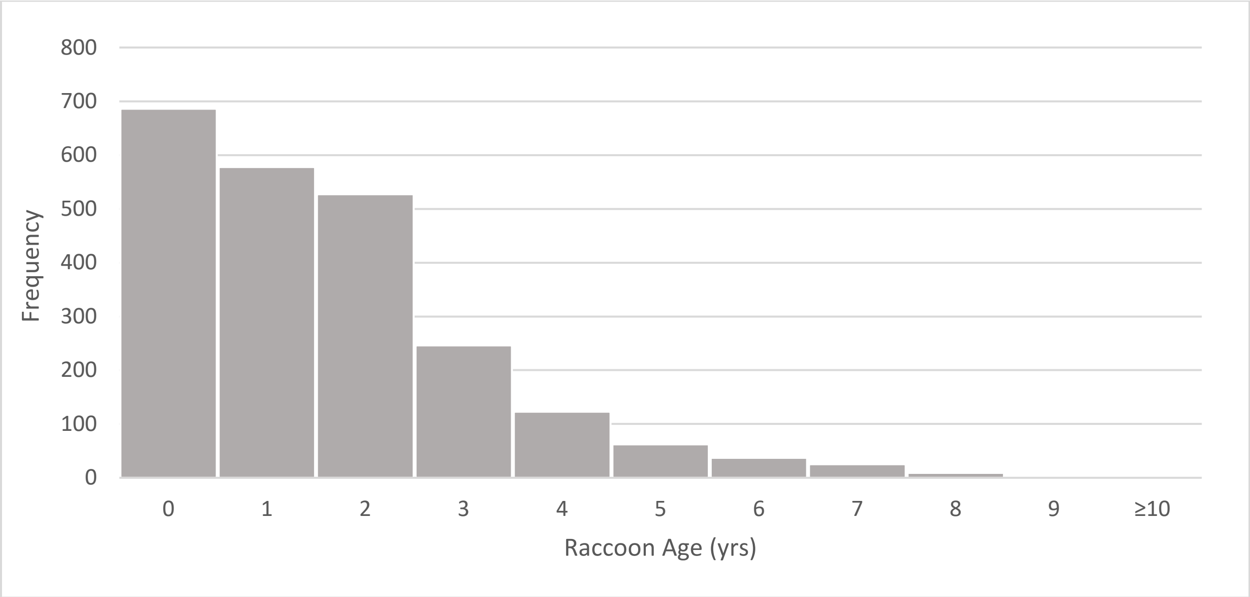

Figure S6: Histogram of raccoon (n=2,296) ages based on cementum annuli of premolar teeth from the oral rabies vaccination (ORV) field trials with Ontario Rabies Vaccine Baits (ONRAB) in West Virginia, USA. The oldest raccoon was 15 years old.

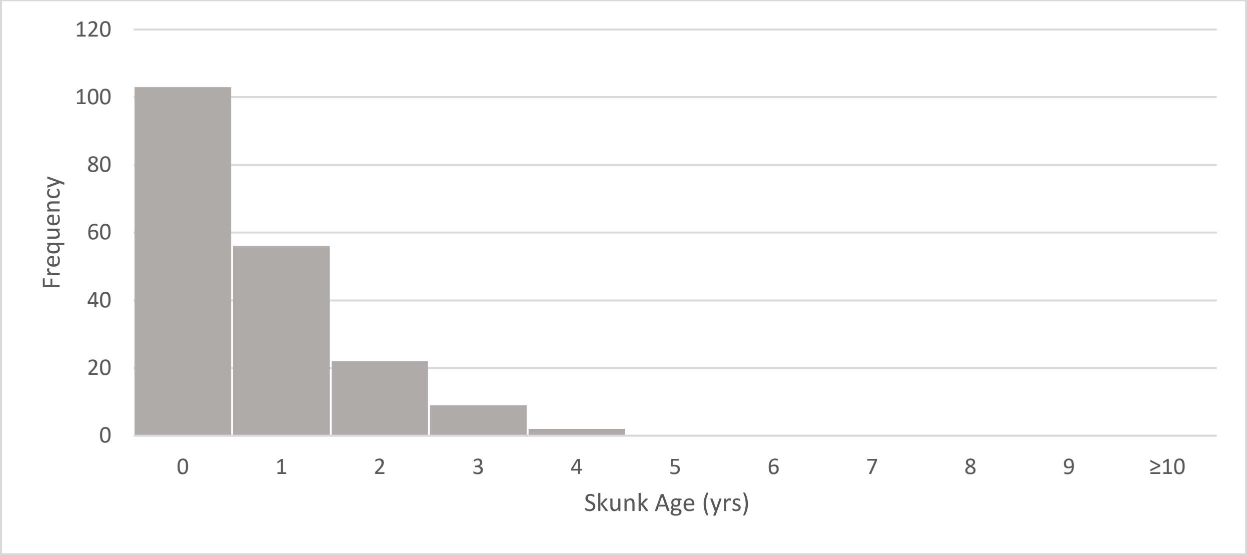

Figure S7: Histogram of skunk (n=192) ages based on cementum annuli of premolar teeth from the oral rabies vaccination (ORV) field trials with Ontario Rabies Vaccine Baits (ONRAB) in West Virginia, USA. The oldest skunk was 4 years old.

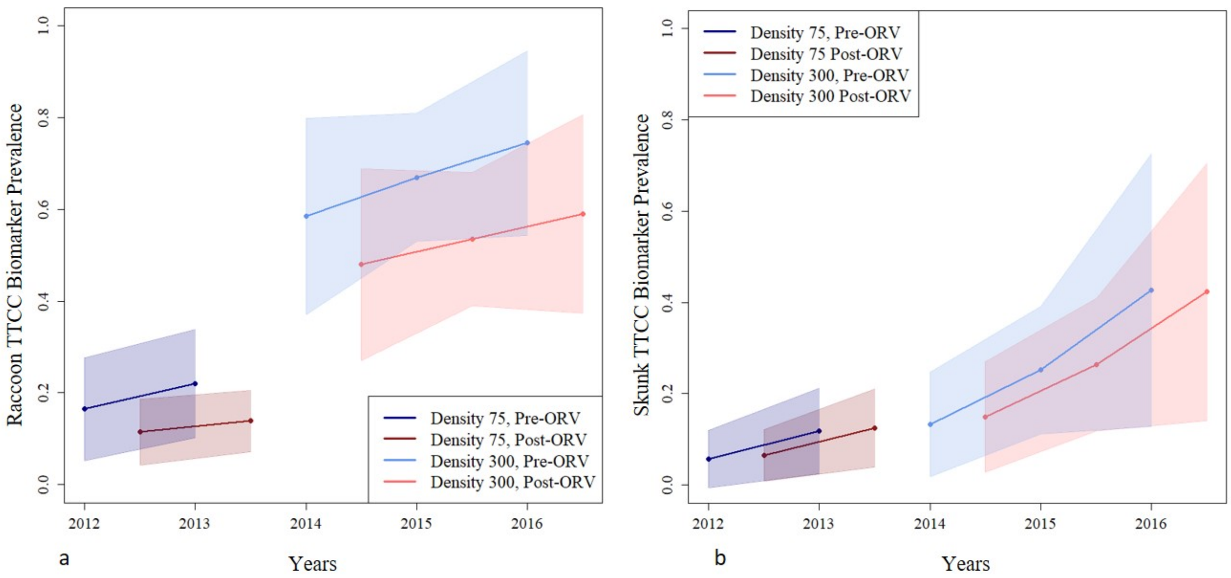

Figure S8: Estimated prevalence of the biomarker tetracycline hydrochloride (TTCC) in raccoon (a) and skunk (b) populations from oral rabies vaccination (ORV) field trials with Ontario Rabies Vaccine Baits (ONRAB) in West Virginia, USA in relationship to bait density, sampling period (pre- or post-ORV), and year. Estimates were from a beta regression analysis and shading represents the 95% confidence interval.

Tables

Table S1: Comparison of rabies virus neutralizing antibody (RVNA) results for a subset of raccoon and skunk serum samples tested at New York State Department of Health (NYSDOH; lab 1) and Kansas State University (lab 2). The RVNA titers were determined from all raccoon and skunk samples at NYSDOH using a cutoff at 0.125 IU/mL. A subset of 300 raccoon samples and 39 skunk samples were tested at lab 2, which used a cutoff of 0.1 IU/mL.

Species	RVNA results at lab 1	Number of samples in study	Number tested by lab 2	Number RVNA positive at lab 2	RVNA agreement between labs 1 and 2
Raccoon (Procyon lotor)					
	<0.125	1053	n.t	n.t	n.t
	0.125	218	100	15	15%
	0.25	278	100	57	57%
	0.5	244	100	91	91%
	>=1	1162	n.t	n.t	n.t
Striped skunks (Mephitis mephitis)					
	<0.125	215	n.t	n.t	n.t
	0.125	18	18	4	22%
	0.25	10	10	8	80%
	0.5	11	11	8	73%
	>=1	36	n.t	n.t	n.t

Table S2: The models compared for the individual level generalized additive modeling analysis for the raccoons and skunks. The same models were used for both species and with three different response variables; which were 1) rabies virus neutralizing antibodies (RVNA) with a seropositive cutoff at 0.125 IU/mL, 2) RVNA at 0.5 IU/mL cutoff, and 3) prevalence of the tetracycline biomarker. Within the structure of the models, "*" indicates both an additive and interaction of the variables surrounding the "*".

Model Name	Predictor Variables and Structure
m001	Period*Sex*Age+Bait Density*Years of Baiting
m002	Period*Sex+Age+Bait Density*Years of Baiting
m003	Period+Sex*Age+Bait Density*Years of Baiting
m004	Period*Age+Sex+Bait Density*Years of Baiting
m005	Period+Sex+Age+Bait Density*Years of Baiting
m006	Period*Sex+Bait Density*Years of Baiting
m007	Period*Age+Bait Density*Years of Baiting
m008	Sex*Age+Bait Density*Years of Baiting
m009	Period+Sex+Bait Density*Years of Baiting
m010	Period+Age+Bait Density*Years of Baiting
m011	Sex+Age+Bait Density*Years of Baiting
m012	Period+Bait Density*Years of Baiting
m013	Sex+Bait Density*Years of Baiting
m014	Age+Bait Density*Years of Baiting
m015	Period*Sex*Age+Bait Density+Years of Baiting
m016	Period*Sex+Age+Bait Density+Years of Baiting
m017	Period+Sex*Age+Bait Density+Years of Baiting
m018	Period*Age+Sex+Bait Density+Years of Baiting
m019	Period+Sex+Age+Bait Density+Years of Baiting
m020	Period*Sex+Bait Density+Years of Baiting
m021	Period*Age+Bait Density+Years of Baiting
m022	Sex*Age+Bait Density+Years of Baiting
m023	Period+Sex+Bait Density+Years of Baiting
m024	Period+Age+Bait Density+Years of Baiting
m025	Sex+Age+Bait Density+Years of Baiting
m026	Period+Bait Density+Years of Baiting
m027	Sex+Bait Density+Years of Baiting
m028	Age+Bait Density+Years of Baiting
m029	Period*Sex*Age+Bait Density
m030	Period*Sex+Age+Bait Density
m031	Period+Sex*Age+Bait Density
m032	Period*Age+Sex+Bait Density
m033	Period+Sex+Age+Bait Density
m034	Period*Sex+Bait Density
m035	Period*Age+Bait Density
m036	Sex*Age+Bait Density
m037	Period+Sex+Bait Density

m038	Period+Age+Bait Density
m039	Sex+Age+Bait Density
m040	Period+Bait Density
m041	Sex+Bait Density
m042	Age+Bait Density
m043	Period*Sex*Age+Years of Baiting
m044	Period*Sex+Age+Years of Baiting
m045	Period+Sex*Age+Years of Baiting
m046	Period*Age+Sex+Years of Baiting
m047	Period+Sex+Age+Years of Baiting
m048	Period*Sex+Years of Baiting
m049	Period*Age+Years of Baiting
m050	Sex*Age+Years of Baiting
m051	Period+Sex+Years of Baiting
m052	Period+Age+Years of Baiting
m053	Sex+Age+Years of Baiting
m054	Period+Years of Baiting
m055	Sex+Years of Baiting
m056	Age+Years of Baiting
m057	Period*Sex*Age
m058	Period*Sex+Age
m059	Period+Sex*Age
m060	Period*Age+Sex
m061	Period+Sex+Age
m062	Period*Sex
m063	Period*Age
m064	Sex*Age
m065	Period+Sex
m066	Period+Age
m067	Sex+Age
m068	Period
m069	Sex
m070	Age
m071	Bait Density*Years of Baiting
m072	Bait Density+Years of Baiting
m073	Bait Density
m074	Years of Baiting
m075	Null

Table S3. Information on target species sampling (n), rabies virus neutralizing antibody (RVNA) seroprevalence (%) at 0.125 IU/mL cutoff and 95% confidence interval for oral rabies vaccination with Ontario Rabies Vaccine Baits (ONRAB) at 75 baits/km² in 2012-2013 and at 300 baits/km² in 2014-2016 in West Virginia, USA. Due to sequential baiting in the cells for the different bait densities, the year 2014 was split; the pre-ORV trapping period had previously been baited at 75 baits/km² and it was only the post-ORV trapping period that followed baiting at 300 baits/km².

	**75 baits/km²**						**300 baits/km²**									
	**2012**			**2013**			**2014**			**2015**			**2016**			
**Species**	**%**	**CI**	**n**	**%**	**CI**	**n**	**%**	**CI**	**n**	**%**	**CI**	**n**	**%**	**CI**	**n**	**Total,n**
Raccoons (Procyon lotor)																
Pre-ORV	41.2	(36.6-45.9)	425	53.3	(48.1-58.4)	351	46.8	(41.0-52.6)	278	81.7	(76.8-85.7)	289	86.9	(82.7-90.2)	320	1663
Post-ORV	55.5	(49.7-61.2)	281	50.7	(44.9-56.5)	284	79.9	(73.5-85.0)	184	82.3	(77.6-86.2)	300	83.1	(77.9-87.3)	243	1292
Striped skunks (Mephitis mephitis)																
Pre-ORV	9	(1.6-37.7)	11	19	(6.6-43.0)	16	10	(3.5-25.6)	30	27.8	(12.5-50.9)	18	31	(12.7-57.6)	13	88
Post-ORV	17	(6.7-35.9)	24	8	(3.3-19.6)	48	46	(32.6-59.7)	48	15.6	(7.8-28.8)	45	60	(43.5-73.7)	37	202
Gray Foxes (Urocyon cinereoargenteus)																
Pre-ORV	-	-	0	-	-	0	0	(0-66)	2	0	(0-79)	1	-	-	0	3
Post-ORV	-	-	0	-	-	0	-	-	0	0	(0-79)	1	-	-	0	1
Red Foxes (Vuples vulpes)																
Pre-ORV	0	(0-66)	2	0	(0-79)	1	-	-	0	0	(0-56)	3	-	-	0	6
Post-ORV	-	-	0	-	-	0	-	-	0	-	-	0	-	-	0	0
Coyotes (Canis latrans)																
Pre-ORV	-	-	0	-	-	0	-	-	0	-	-	0	-	-	0	0
Post-ORV	0	(0-79.4)	1	-	-	0	-	-	0	-	-	0	-	-	0	1

Table S4. Information on target species sampling (n), rabies virus neutralizing antibody (RVNA) seroprevalence (%) at the 0.5 IU/ml cutoff and 95% confidence interval for oral rabies vaccination with Ontario Rabies Vaccine Baits (ONRAB) at 75 baits/km^2 in 2012-2013 and at 300 baits/km^2 in 2014-2016 in West Virginia, USA. Due to sequential baiting in the cells for the different bait densities, the year 2014 was split; the pre-ORV trapping period had previously been baited at 75 baits/km^2 and it was only the post-ORV trapping period that followed baiting at 300 baits/km^2. All sampled coyotes (n=1) and foxes (n=10) were negative at 0.5 IU/ml and are not included in this table.

	75 baits/km2						300 baits/km2									
	2012			2013			2014			2015			2016			
Species	%	CI	n	%	CI	n	%	CI	n	%	CI	n	%	CI	n	Total n
Raccoons (Procyon lotor)																
Pre-ORV	16.7	(13.5-20.6)	425	27.3	(23.0-32.2)	351	29.9	(24.8-35.5)	278	66.8	(61.2-72.0)	289	67.5	(62.2-72.4)	320	1663
Post-ORV	45.2	(39.5-51.0)	281	38	(32.6-43.8)	284	71.2	(64.2-77.3)	184	71.7	(66.3-76.5)	300	68.3	(62.2-73.8)	243	1292
Striped skunks (Mephitis mephitis)																
Pre-ORV	0	(0-25.9)	11	0	(0-19.4)	16	3	(0.6-16.7)	30	17	(5.8-39.2)	18	8	(1.4-33.3)	13	88
Post-ORV	17	(6.7-35.9)	24	4	(1.2-14.0)	48	25	(14.9-38.8)	48	13	(6.3-26.2)	45	49	(33.5-64.1)	37	202

Table S5: Covariate estimates for generalized linear mixed model on raccoon and skunk seroprevalence from oral rabies vaccination (ORV) at the 0.5 IU/mL cutoff in West Virginia, USA.

Species	Parameter	Estimate	Std. Error	z value	Pr(>|z|)
Raccoons	Intercept	-3.23	0.40	-8.15	0
	Bait density	0.01	0.00	9.21	0
	Years of baiting	0.66	0.14	4.74	0
	Period (pre- or post-ORV)	2.40	0.47	5.11	0
	Years of baiting X Period	-0.71	0.20	-3.56	0
Skunks	Intercept	-4.24	0.85	-5.01	0.00
	Bait density	0.01	0.00	3.58	0.00
	Years of baiting	0.21	0.28	0.76	0.45
	Period (pre- or post-ORV)	1.29	0.87	1.49	0.14
	Years of baiting X Period	-0.05	0.37	-0.12	0.90

Table S6: Model selection table of the individual level generalized additive modeling candidate model set for raccoon seroprevalence at the 0.125 IU/mL cutoff. Models are sorted from most parsimonious on top to least parsimonious based on Akaike's information criterion corrected (AICc) for small sample sizes. The model names, degrees of freedom (df), the log likelihood (LL), the AICc, the delta AICc (difference between the model AICc value and the top model), the model likelihood(ModelLik), the AICc weight (relative support for each model), and the cumulative AICc weight (Cum.Wt, representing total cumulative weight for the given model and all models above it) are shown.

Model Name	df	LL	AICc	delta AICc	Cum. Wt
m004	10	-1646.705	3314.802	0.00000	0.6516
m007	9	-1648.785	3316.945	2.14299	0.2232
m001	13	-1645.445	3318.324	3.52216	0.1120
m018	9	-1652.552	3324.488	9.68593	0.0051
m021	8	-1654.430	3326.229	11.42713	0.0022
m005	9	-1653.456	3326.290	11.48809	0.0021
m003	10	-1652.893	3327.178	12.37588	0.0013
m015	12	-1651.184	3327.787	12.98427	0.0010
m002	10	-1653.389	3328.171	13.36842	0.0008
m010	8	-1655.588	3328.538	13.73564	0.0007
m019	8	-1659.807	3336.983	22.18119	0.0000
m017	9	-1659.263	3337.907	23.10501	0.0000
m024	7	-1661.716	3338.788	23.98600	0.0000
m016	9	-1659.712	3338.805	24.00316	0.0000
m011	8	-1686.354	3390.039	75.23625	0.0000
m014	7	-1687.913	3391.138	76.33585	0.0000
m008	9	-1686.059	3391.460	76.65783	0.0000
m025	7	-1692.378	3400.082	85.27959	0.0000
m028	6	-1693.732	3400.776	85.97416	0.0000
m032	8	-1691.800	3401.057	86.25447	0.0000
m022	8	-1692.107	3401.551	86.74916	0.0000
m035	7	-1693.711	3402.870	88.06720	0.0000
m029	11	-1690.292	3404.073	89.27091	0.0000
m033	7	-1698.040	3411.526	96.72358	0.0000
m031	8	-1697.304	3412.064	97.26217	0.0000
m030	8	-1697.972	3413.401	98.59897	0.0000
m038	6	-1699.982	3413.402	98.59940	0.0000
m039	6	-1732.309	3478.040	163.23785	0.0000
m042	5	-1733.696	3478.805	164.00277	0.0000
m036	7	-1731.887	3479.206	164.40350	0.0000
m012	7	-1738.904	3493.183	178.38044	0.0000
m009	8	-1738.075	3493.539	178.73659	0.0000
m006	9	-1738.041	3495.485	180.68282	0.0000
m026	6	-1745.910	3505.183	190.38051	0.0000
m023	7	-1745.238	3505.850	191.04796	0.0000
m020	8	-1745.179	3507.745	192.94230	0.0000

m071	6	-1749.563	3512.459	197.65638	0.0000
m013	7	-1748.855	3513.056	198.25336	0.0000
m072	5	-1756.268	3523.860	209.05723	0.0000
m027	6	-1755.703	3524.740	209.93772	0.0000
m046	8	-1775.054	3568.154	253.35122	0.0000
m043	11	-1773.923	3571.937	257.13452	0.0000
m049	7	-1778.300	3572.634	257.83136	0.0000
m040	5	-1781.838	3575.097	260.29503	0.0000
m037	6	-1781.086	3575.601	260.79890	0.0000
m034	7	-1781.048	3577.534	262.73190	0.0000
m045	8	-1780.752	3579.550	264.74733	0.0000
m047	7	-1781.849	3579.732	264.92920	0.0000
m044	8	-1781.672	3581.389	266.58680	0.0000
m052	6	-1785.228	3584.479	269.67651	0.0000
m060	7	-1784.331	3584.692	269.88954	0.0000
m057	10	-1783.099	3588.270	273.46751	0.0000
m063	6	-1787.431	3588.881	274.07907	0.0000
m059	7	-1789.461	3594.953	280.15071	0.0000
m061	6	-1790.612	3595.243	280.44049	0.0000
m073	4	-1793.310	3596.017	281.21445	0.0000
m041	5	-1792.680	3596.763	281.96026	0.0000
m058	7	-1790.447	3596.924	282.12160	0.0000
m066	5	-1793.836	3599.683	284.88045	0.0000
m053	6	-1817.450	3648.924	334.12148	0.0000
m050	7	-1816.730	3649.495	334.69310	0.0000
m056	5	-1820.074	3652.163	337.36055	0.0000
m067	5	-1826.698	3665.407	350.60508	0.0000
m064	6	-1825.935	3665.890	351.08774	0.0000
m070	4	-1829.180	3668.363	353.56048	0.0000
m051	6	-1870.576	3755.176	440.37323	0.0000
m054	5	-1872.307	3756.627	441.82503	0.0000
m048	7	-1870.504	3757.041	442.23902	0.0000
m065	5	-1877.738	3767.488	452.68558	0.0000
m068	4	-1879.390	3768.782	453.98017	0.0000
m062	6	-1877.672	3769.364	454.56134	0.0000
m055	5	-1882.483	3776.980	462.17725	0.0000
m074	4	-1883.993	3777.992	463.18962	0.0000
m069	4	-1889.978	3789.960	475.15736	0.0000
m075	3	-1891.412	3790.821	476.01834	0.0000

Table S7: Model selection table of the individual level generalized additive modeling candidate model set for skunk seroprevalence at the 0.125 IU/mL cutoff. Models are sorted from most parsimonious on top to least parsimonious based on Akaike's information criterion corrected (AICc) for small sample sizes. The model names, degrees of freedom (df), the log likelihood (LL), the AICc, the delta AICc (difference between the model AICc value and the top model), the model likelihood(ModelLik), the AICc weight (relative support for each model), and the cumulative AICc weight (Cum.Wt, representing total cumulative weight for the given model and all models above it) are shown.

Model Name	df	LL	AICc	delta AICc	Cum. Wt
m026	4	-153.970	316.080	0.00000	0.0993
m040	3	-155.131	316.346	0.26571	0.0869
m038	4	-154.138	316.417	0.33730	0.0839
m024	5	-153.194	316.600	0.51967	0.0765
m012	5	-153.446	317.104	1.02400	0.0595
m010	6	-152.560	317.416	1.33655	0.0509
m023	5	-153.938	318.088	2.00834	0.0364
m035	5	-153.967	318.145	2.06525	0.0353
m021	6	-152.969	318.234	2.15464	0.0338
m037	4	-155.100	318.340	2.25995	0.0321
m033	5	-154.089	318.390	2.30992	0.0313
m072	3	-156.165	318.413	2.33351	0.0309
m019	6	-153.144	318.586	2.50578	0.0284
m009	6	-153.377	319.052	2.97211	0.0225
m007	7	-152.335	319.067	2.98722	0.0223
m005	7	-152.458	319.314	3.23390	0.0197
m073	2	-157.671	319.384	3.30396	0.0190
m071	4	-155.647	319.434	3.35384	0.0186
m028	4	-155.762	319.665	3.58564	0.0165
m032	6	-153.911	320.119	4.03886	0.0132
m020	6	-153.935	320.166	4.08636	0.0129
m018	7	-152.912	320.221	4.14133	0.0125
m042	3	-157.085	320.254	4.17393	0.0123
m027	4	-156.096	320.333	4.25299	0.0118
m034	5	-155.099	320.409	4.32931	0.0114
m031	6	-154.078	320.453	4.37274	0.0111
m030	6	-154.086	320.468	4.38862	0.0111
m014	5	-155.159	320.530	4.44998	0.0107
m016	7	-153.137	320.672	4.59238	0.0100
m017	7	-153.144	320.685	4.60513	0.0099
m004	8	-152.225	320.963	4.88277	0.0086
m006	7	-153.365	321.128	5.04783	0.0080
m013	5	-155.533	321.277	5.19694	0.0074
m041	3	-157.597	321.278	5.19768	0.0074
m002	8	-152.439	321.391	5.31109	0.0070
m003	8	-152.458	321.429	5.34898	0.0068

m025	5	-155.674	321.559	5.47947	0.0064
m039	4	-156.990	322.121	6.04099	0.0048
m011	6	-155.014	322.325	6.24477	0.0044
m022	6	-155.673	323.644	7.56424	0.0023
m036	5	-156.959	324.130	8.05002	0.0018
m008	7	-155.013	324.424	8.34372	0.0015
m029	9	-153.231	325.105	9.02543	0.0011
m015	10	-152.257	325.303	9.22358	0.0010
m001	11	-151.529	326.007	9.92735	0.0007
m066	3	-162.193	331.393	15.31291	0.0000
m068	2	-163.713	331.523	15.44320	0.0000
m063	4	-161.869	332.914	16.83420	0.0000
m052	4	-161.792	333.105	17.02503	0.0000
m061	4	-162.169	333.279	17.19941	0.0000
m065	3	-163.658	333.409	17.32895	0.0000
m054	3	-163.510	333.437	17.35753	0.0000
m075	1	-165.157	333.746	17.66665	0.0000
m070	2	-164.108	334.266	18.18575	0.0000
m049	5	-161.523	334.694	18.61374	0.0000
m060	5	-161.835	334.790	18.70981	0.0000
m059	5	-162.139	334.891	18.81121	0.0000
m047	5	-161.763	335.021	18.94088	0.0000
m058	5	-162.103	335.193	19.11340	0.0000
m051	4	-163.498	335.308	19.22822	0.0000
m062	4	-163.604	335.357	19.27694	0.0000
m069	2	-165.125	335.583	19.50356	0.0000
m074	2	-165.055	335.718	19.63830	0.0000
m056	4	-163.906	336.124	20.04380	0.0000
m067	3	-164.062	336.130	20.05029	0.0000
m046	6	-161.486	336.599	20.51941	0.0000
m045	6	-161.704	336.654	20.57409	0.0000
m044	6	-161.715	336.978	20.89771	0.0000
m048	5	-163.463	337.277	21.19708	0.0000
m055	3	-165.019	337.571	21.49164	0.0000
m064	4	-163.994	337.716	21.63603	0.0000
m053	5	-163.856	338.005	21.92500	0.0000
m057	8	-161.143	339.418	23.33767	0.0000
m050	5	-163.763	339.599	23.51934	0.0000
m043	9	-160.756	341.246	25.16569	0.0000

Table S8: Parameters and estimates of the top competitive models for the individual level generalized additive modeling for raccoons based on a rabies virus neutralizing antibody (RVNA) cutoff of 0.125 IU/mL. Oral rabies vaccination (ORV) occurred with Ontario Rabies Vaccine Baits (ONRAB) in West Virginia, USA.

	**Model Name**		
	**m04**		
**Parameter**	**Estimate (SE)**		
Intercept	-0.41765	**	(0.16)
Period Post-ORV	-0.07669		(0.14)
Sex	0.17877		(0.09)
Age	0.30068	***	(0.04)
Bait Density 75	-0.80308	***	(0.20)
Years of Baiting	0.69821	***	(0.07)
Period Post-ORV: Sex	--		--
Period Post-ORV: Age	0.26945	***	(0.07)
Sex:Age	--		--
Bait Density 75: Years of Baiting	-0.40882	***	(0.11)
Period Post-ORV: Sex: Age	--		--

Significant codes: 0 ‘***’ 0.001 ‘**’ 0.01 ‘*’ 0.05 ‘.’ 0.1 ‘ ’ 1

Table S9: Parameters and estimates of the top competitive models for the individual level generalized additive modeling for skunks based on a rabies virus neutralizing antibody (RVNA) cutoff of 0.125 IU/mL. Oral rabies vaccination (ORV) occurred with Ontario Rabies Vaccine Baits (ONRAB) in West Virginia, USA.

	**Model Name**								
	**m026**			**m040**			**m038**		
**Parameter**	**Estimate (SE)**			**Estimate (SE)**			**Estimate (SE)**		
Intercept	-1.7153	***	(0.45)	-1.2102	***	(0.29)	-1.4655	***	(0.34)
Period Post-ORV	0.6642	*	(0.33)	0.7073	*	(0.32)	0.7713	*	(0.33)
Sex	--		--	--		--	--		--
Age	--		--	--		--	0.2271		(0.16)
Bait Density 75	-1.5391	***	(0.38)	-1.3205	***	(0.35)	-1.3106	***	(0.35)
Years of Baiting	0.2836		(0.19)	--		--	--		--
Period Post-ORV: Sex	--		--	--		--	--		--
Period Post-ORV: Age	--		--	--		--	--		--
Sex:Age	--		--	--		--	--		--
Bait Density 75: Years of Baiting	--		--	--		--	--		--
Period Post-ORV: Sex: Age	--		--	--		--	--		--

	**Model Name**								
	**m024**			**m012**			**m10**		
**Parameter**	**Estimate (SE)**			**Estimate (SE)**			**Estimate (SE)**		
Intercept	-1.9008	***	(0.48)	-1.8246	***	(0.46)	- 2.0356	***	(0.49)
Period Post-ORV	0.7289	*	(0.33)	0.6656	*	(0.33)	0.7341	*	(0.33)
Sex	--		--	--		--	--		--
Age	0.203		(0.16)	--		--	0.2184		(0.16)
Bait Density 75	-1.5136	***	(0.38)	0.235		(1.71)	0.4596		(1.72)
Years of Baiting	0.2582		(0.19)	0.3398	.	(0.19)	0.3187		(0.20)
Period Post-ORV: Sex	--		--	--		--	--		--
Period Post-ORV: Age	--		--	--		--	--		--
Sex:Age	--		--	--		--	--		--
Bait Density 75: Years of Baiting	--		--	-0.6854		(0.66)	- 0.7601		(0.66)
Period Post-ORV: Sex: Age	--		--	--		--	--		--

Significant codes: 0 ‘***’ 0.001 ‘**’ 0.01 ‘*’ 0.05 ‘.’ 0.1 ‘ ’ 1

Table S10: Covariate estimates for generalized linear mixed model on portion of the tetracycline marked raccoons and skunks from oral rabies vaccination (ORV) in West Virginia, USA.

Species	Parameter	Estimate	Std. Error	z value	Pr(>|z|)
Raccoons	Intercept	-3.96	0.85	-4.63	0.00
	Bait density	3.94	0.76	5.19	0.00
	Years of baiting	0.36	0.38	0.96	0.34
	Period (pre- or post-ORV)	-0.28	0.92	-0.31	0.76
	Years of baiting X Period	-0.14	0.48	-0.29	0.77
Skunks	Intercept	-4.52	1.15	-3.94	0.00
	Bait density	1.85	0.82	2.26	0.02
	Years of baiting	0.79	0.43	1.86	0.06
	Period (pre- or post-ORV)	0.21	1.04	0.20	0.84
	Years of baiting X Period	-0.08	0.56	-0.14	0.89

Table S11: The models compared for the generalized liner mixed model analysis for the raccoons and skunks. The same models were used for both species and with two different response variables; which were 1) rabies virus neutralizing antibodies (RVNA) with a seropositive cutoff at 0.125 IU/mL and 2) RVNA at 0.5 IU/mL cutoff. Within the structure of the models, "*" indicates both an additive and interaction of the variables surrounding the "*".

Model Name	Predictor Variables and Structure
Density_YearByPeriod	Density + Years of Baiting*Period
Density_Year_Period	Density + Years of Baiting + Period
Density_Period	Density + Period
Density_Year	Density + Years of Baiting
Year_Period	Years of Baiting + Period
Density	Density
Period	Period
Year	Years of Baiting
Null	Null

Table S12: Model selection table of the generalized liner mixed candidate model set for raccoon seroprevalence at the 0.125 IU/mL cutoff. Models are sorted from most parsimonious on top to least parsimonious based on Akaike's information criterion corrected (AICc) for small sample sizes. The model names, number of parameters (*k*), the AICc, the delta AICc (difference between the model AICc value and the top model), the model likelihood(ModelLik), the AICc weight (relative support for each model), the log likelihood (LL), and the cumulative AICc weight (Cum.Wt, representing total cumulative weight for the given model and all models above it) are shown.

Model Name	K	AICc	Delta_AICc	ModelLik	AICcWt	LL	Cum.Wt
Density_YearByPeriod	7	-55.97	0.00	1.00	0.99	37.14	0.99
Density_Year_Period	6	-45.67	10.30	0.01	0.01	30.39	1.00
Density_Year	5	-44.00	11.97	0.00	0.00	28.07	1.00
Density	4	-38.67	17.30	0.00	0.00	24.03	1.00
Density_Period	5	-38.20	17.78	0.00	0.00	25.17	1.00
Null	3	-16.72	39.25	0.00	0.00	11.76	1.00
Period	4	-15.25	40.72	0.00	0.00	12.31	1.00
Year	4	-14.41	41.57	0.00	0.00	11.89	1.00
Year_Period	5	-12.86	43.11	0.00	0.00	12.50	1.00

Table S13: Model selection table of the generalized liner mixed candidate model set for raccoon seroprevalence at the 0.5 IU/mL cutoff. Models are sorted from most parsimonious on top to least parsimonious based on Akaike's information criterion corrected (AICc) for small sample sizes. The model names, number of parameters (*k*), the AICc, the delta AICc (difference between the model AICc value and the top model), the model likelihood(ModelLik), the AICc weight (relative support for each model), the log likelihood (LL), and the cumulative AICc weight (Cum.Wt, representing total cumulative weight for the given model and all models above it) are shown.

Model Name	K	AICc	Delta_AICc	ModelLik	AICcWt	LL	Cum.Wt
Density_YearByPeriod	7	-50.12	0.00	1.00	0.98	34.21	0.98
Density_Year_Period	6	-42.20	7.92	0.02	0.02	28.65	1.00
Density_Period	5	-38.77	11.35	0.00	0.00	25.46	1.00
Density_Year	5	-27.57	22.55	0.00	0.00	19.86	1.00
Density	4	-26.93	23.19	0.00	0.00	18.16	1.00
Period	4	-11.23	38.89	0.00	0.00	10.30	1.00
Year_Period	5	-8.51	41.61	0.00	0.00	10.33	1.00
Null	3	-7.01	43.11	0.00	0.00	6.91	1.00
Year	4	-4.46	45.65	0.00	0.00	6.92	1.00

Table S14: Model selection table of the generalized liner mixed candidate model set for skunk seroprevalence at the 0.125 IU/mL cutoff. Models are sorted from most parsimonious on top to least parsimonious based on Akaike's information criterion corrected (AICc) for small sample sizes. The model names, number of parameters (*k*), the AICc, the delta AICc (difference between the model AICc value and the top model), the model likelihood(ModelLik), the AICc weight (relative support for each model), the log likelihood (LL), and the cumulative AICc weight (Cum.Wt, representing total cumulative weight for the given model and all models above it) are shown.

Model Name	K	AICc	Delta_AICc	ModelLik	AICcWt	LL	Cum.Wt
Density_Year	5	-28.40	0.00	1.00	0.32	20.31	0.32
Density	4	-27.93	0.47	0.79	0.25	18.68	0.58
Density_Year_Period	6	-26.84	1.56	0.46	0.15	21.04	0.72
Density_Period	5	-26.78	1.62	0.45	0.14	19.50	0.87
Density_YearByPeriod	7	-25.96	2.43	0.30	0.10	22.22	0.96
Null	3	-22.96	5.44	0.07	0.02	14.89	0.98
Period	4	-20.94	7.46	0.02	0.01	15.18	0.99
Year	4	-20.45	7.95	0.02	0.01	14.94	1.00
Year_Period	5	-18.20	10.20	0.01	0.00	15.21	1.00

Table S15: Model selection table of the generalized liner mixed candidate model set for skunk seroprevalence at the 0.5 IU/mL cutoff. Models are sorted from most parsimonious on top to least parsimonious based on Akaike's information criterion corrected (AICc) for small sample sizes. The model names, number of parameters (*k*), the AICc, the delta AICc (difference between the model AICc value and the top model), the model likelihood(ModelLik), the AICc weight (relative support for each model), the log likelihood (LL), and the cumulative AICc weight (Cum.Wt, representing total cumulative weight for the given model and all models above it) are shown.

Model Name	K	AICc	Delta_AICc	ModelLik	AICcWt	LL	Cum.Wt
Density_Period	5	-75.49	0.00	1.00	0.69	43.86	0.69
Density_Year_Period	6	-73.35	2.14	0.34	0.24	44.29	0.93
Density_YearByPeriod	7	-70.11	5.38	0.07	0.05	44.30	0.98
Period	4	-67.14	8.36	0.02	0.01	38.28	0.99
Density	4	-65.66	9.84	0.01	0.01	37.54	0.99
Year_Period	5	-65.09	10.41	0.01	0.00	38.65	1.00
Density_Year	5	-63.16	12.33	0.00	0.00	37.69	1.00
Null	3	-62.73	12.76	0.00	0.00	34.78	1.00
Year	4	-60.55	14.94	0.00	0.00	34.99	1.00

Table S16: Model selection table of the individual level generalized additive modeling candidate model set for raccoon seroprevalence at the 0.5 IU/mL cutoff. Models are sorted from most parsimonious on top to least parsimonious based on Akaike's information criterion corrected (AICc) for small sample sizes (AICc). The model names, degrees of freedom (df), the log likelihood (LL), the AICc, the delta AICc (difference between the model AICc value and the top model), the model likelihood(ModelLik), the AICc weight (relative support for each model), and the cumulative AICc weight (Cum.Wt, representing total cumulative weight for the given model and all models above it) are shown.

Model Name	df	LL	AICc	delta AICc	Cum. Wt
m004	10	-1709.287	3440.137	0.00000	0.3648
m018	9	-1710.804	3441.148	1.01105	0.2200
m001	13	-1707.033	3441.706	1.56864	0.1665
m007	9	-1711.665	3442.866	2.72903	0.0932
m015	12	-1708.690	3442.992	2.85450	0.0875
m021	8	-1713.048	3443.613	3.47531	0.0642
m005	9	-1716.240	3452.016	11.87880	0.0010
m002	10	-1715.274	3452.107	11.96994	0.0009
m019	8	-1717.985	3453.483	13.34576	0.0005
m003	10	-1716.028	3453.609	13.47179	0.0004
m016	9	-1717.091	3453.716	13.57848	0.0004
m010	8	-1718.761	3455.032	14.89521	0.0002
m017	9	-1717.791	3455.110	14.97232	0.0002
m024	7	-1720.359	3456.210	16.07264	0.0001
m032	8	-1734.017	3485.601	45.46425	0.0000
m029	11	-1731.653	3486.938	46.80123	0.0000
m035	7	-1736.331	3488.208	48.07103	0.0000
m033	7	-1740.616	3496.777	56.64022	0.0000
m030	8	-1739.684	3496.932	56.79504	0.0000
m031	8	-1740.316	3498.192	58.05456	0.0000
m038	6	-1743.055	3499.636	59.49889	0.0000
m006	9	-1803.142	3625.801	185.66344	0.0000
m012	7	-1805.252	3625.988	185.85104	0.0000
m009	8	-1804.370	3626.240	186.10240	0.0000
m026	6	-1807.353	3628.172	188.03481	0.0000
m020	8	-1805.423	3628.341	188.20365	0.0000
m011	8	-1805.503	3628.491	188.35419	0.0000
m023	7	-1806.572	3628.621	188.48398	0.0000
m014	7	-1806.801	3629.068	188.93054	0.0000
m025	7	-1806.902	3629.271	189.13333	0.0000
m028	6	-1808.102	3629.655	189.51780	0.0000
m008	9	-1805.495	3630.487	190.35009	0.0000
m022	8	-1806.896	3631.272	191.13484	0.0000
m040	5	-1827.914	3667.316	227.17880	0.0000
m034	7	-1825.911	3667.334	227.19722	0.0000
m037	6	-1827.090	3667.679	227.54133	0.0000

m039	6	-1830.391	3674.292	234.15491	0.0000
m042	5	-1831.610	3674.716	234.57900	0.0000
m036	7	-1830.360	3676.241	236.10345	0.0000
m046	8	-1851.519	3721.090	280.95227	0.0000
m043	11	-1848.845	3721.787	281.65022	0.0000
m071	6	-1854.763	3722.969	282.83150	0.0000
m060	7	-1853.877	3723.794	283.65657	0.0000
m013	7	-1854.199	3723.853	283.71592	0.0000
m072	5	-1856.367	3724.163	284.02543	0.0000
m057	10	-1851.202	3724.485	284.34745	0.0000
m027	6	-1855.868	3725.176	285.03906	0.0000
m049	7	-1855.431	3726.902	286.76446	0.0000
m063	6	-1857.755	3729.540	289.40237	0.0000
m047	7	-1858.728	3733.496	293.35919	0.0000
m044	8	-1858.126	3734.305	294.16742	0.0000
m045	8	-1858.210	3734.472	294.33508	0.0000
m061	6	-1860.798	3735.624	295.48675	0.0000
m058	7	-1860.191	3736.422	296.28508	0.0000
m059	7	-1860.259	3736.558	296.42028	0.0000
m052	6	-1862.842	3739.713	299.57534	0.0000
m066	5	-1864.876	3741.770	301.63309	0.0000
m073	4	-1878.052	3765.565	325.42823	0.0000
m041	5	-1877.537	3766.544	326.40702	0.0000
m053	6	-1947.685	3909.399	469.26142	0.0000
m051	6	-1948.015	3910.058	469.92059	0.0000
m048	7	-1947.149	3910.337	470.19995	0.0000
m065	5	-1949.411	3910.841	470.70416	0.0000
m062	6	-1948.540	3911.109	470.97152	0.0000
m050	7	-1947.577	3911.194	471.05645	0.0000
m054	5	-1949.952	3911.924	471.78664	0.0000
m067	5	-1950.057	3912.134	471.99648	0.0000
m056	5	-1950.249	3912.518	472.38121	0.0000
m068	4	-1951.328	3912.666	472.52876	0.0000
m064	6	-1949.939	3913.906	473.76841	0.0000
m070	4	-1952.582	3915.174	475.03696	0.0000
m055	5	-1997.503	4007.026	566.88830	0.0000
m074	4	-1998.920	4007.851	567.71327	0.0000
m069	4	-1999.218	4008.447	568.31002	0.0000
m075	3	-2000.612	4009.229	569.09163	0.0000

Table S17: Model selection table of the individual level generalized additive modeling candidate model set for skunk seroprevalence at the 0.5 IU/mL cutoff. Models are sorted from most parsimonious on top to least parsimonious based on Akaike's information criterion corrected (AICc) for small sample sizes. The model names, degrees of freedom (df), the log likelihood (LL), the AICc, the delta AICc (difference between the model AICc value and the top model), the model likelihood(ModelLik), the AICc weight (relative support for each model), and the cumulative AICc weight (Cum.Wt, representing total cumulative weight for the given model and all models above it) are shown.

Model Name	df	LL	AICc	delta AICc	Cum. Wt
m010	6	-108.123	228.544	0.00000	0.3137
m007	7	-107.963	230.323	1.77867	0.1289
m005	7	-108.036	230.470	1.92575	0.1198
m024	5	-110.984	232.179	3.63480	0.0510
m012	5	-110.990	232.192	3.64744	0.0506
m002	8	-107.855	232.222	3.67813	0.0499
m004	8	-107.872	232.257	3.71301	0.0490
m003	8	-108.036	232.584	4.03995	0.0416
m038	4	-111.998	232.916	4.37145	0.0353
m021	6	-110.828	233.953	5.40886	0.0210
m009	6	-110.967	234.231	5.68673	0.0183
m019	6	-110.975	234.247	5.70279	0.0181
m035	5	-111.870	234.748	6.20362	0.0141
m033	5	-112.001	234.980	6.43603	0.0126
m026	4	-113.478	235.096	6.55228	0.0119
m006	7	-110.809	236.015	7.47057	0.0075
m018	7	-110.817	236.032	7.48745	0.0074
m016	7	-110.853	236.104	7.55997	0.0072
m017	7	-110.975	236.347	7.80304	0.0063
m040	3	-115.160	236.406	7.86163	0.0062
m032	6	-111.875	236.824	8.27948	0.0050
m030	6	-111.904	236.853	8.30903	0.0049
m031	6	-112.013	236.998	8.45367	0.0046
m023	5	-113.477	237.165	8.62116	0.0042
m001	11	-107.533	238.016	9.47191	0.0028
m037	4	-115.157	238.456	9.91184	0.0022
m020	6	-113.374	239.045	10.50062	0.0016
m034	5	-115.077	240.367	11.82319	0.0008
m014	5	-115.660	241.534	12.98968	0.0005
m066	5	-115.638	241.703	13.15860	0.0004
m015	10	-110.527	241.844	13.29981	0.0004
m071	4	-117.237	242.616	14.07143	0.0003
m029	9	-111.674	242.635	14.09107	0.0003
m063	6	-115.367	243.311	14.76694	0.0002
m011	6	-115.525	243.350	14.80551	0.0002
m052	6	-115.584	243.664	15.11971	0.0002

m061	6	-115.635	243.775	15.23120	0.0002
m013	5	-117.171	244.554	16.00952	0.0001
m028	4	-118.382	244.908	16.36346	0.0001
m049	7	-115.328	245.311	16.76713	0.0001
m060	7	-115.362	245.395	16.85061	0.0001
m072	3	-119.663	245.410	16.86586	0.0001
m058	7	-115.422	245.436	16.89206	0.0001
m008	7	-115.522	245.443	16.89916	0.0001
m047	7	-115.582	245.752	17.20831	0.0001
m059	7	-115.618	245.763	17.21853	0.0001
m042	4	-118.764	246.269	17.72479	0.0000
m025	5	-118.338	246.890	18.34581	0.0000
m046	8	-115.324	247.411	18.86693	0.0000
m068	3	-119.796	247.429	18.88526	0.0000
m027	4	-119.649	247.439	18.89530	0.0000
m044	8	-115.379	247.448	18.90353	0.0000
m045	8	-115.556	247.738	19.19361	0.0000
m073	2	-121.030	247.987	19.44333	0.0000
m039	5	-118.747	248.281	19.73701	0.0000
m022	6	-118.334	248.967	20.42303	0.0000
m054	4	-119.796	249.480	20.93615	0.0000
m065	4	-119.799	249.495	20.95081	0.0000
m041	3	-121.036	250.019	21.47457	0.0000
m036	6	-118.756	250.294	21.74949	0.0000
m057	10	-114.999	250.993	22.44859	0.0000
m062	5	-119.630	251.236	22.69175	0.0000
m051	5	-119.799	251.560	23.01612	0.0000
m043	11	-114.969	253.068	24.52410	0.0000
m070	4	-122.348	253.251	24.70701	0.0000
m048	6	-119.631	253.317	24.77268	0.0000
m067	5	-122.338	255.296	26.75221	0.0000
m056	5	-122.351	255.310	26.76536	0.0000
m075	3	-124.859	255.988	27.44399	0.0000
m064	6	-122.273	257.166	28.62224	0.0000
m053	6	-122.341	257.369	28.82490	0.0000
m074	4	-124.823	257.956	29.41180	0.0000
m069	4	-124.860	258.037	29.49234	0.0000
m050	7	-122.274	259.250	30.70614	0.0000
m055	5	-124.825	260.020	31.47535	0.0000

Table S18: Parameters and estimates of the top competitive models for the individual level generalized additive modeling for raccoons based on a rabies virus neutralizing antibody (RVNA) cutoff at 0.5 IU/mL. Oral rabies vaccination (ORV) occurred with Ontario Rabies Vaccine Baits (ONRAB) in West Virginia, USA.

	**Model Name**								
	**m004**			**m018**			**m001**		
**Parameter**	**Estimate (SE)**			**Estimate (SE)**			**Estimate (SE)**		
Intercept	-1.51944	***	(0.20)	-1.40042	***	(0.19)	-1.3683	***	(0.22)
Period Post-ORV	0.8292	***	(0.13)	0.82252	***	(0.13)	0.63591	***	(0.19)
Sex	0.1838	*	(0.08)	0.17843	*	(0.08)	-0.08784		(0.19)
Age	0.28737	***	(0.04)	0.28699	***	(0.04)	0.25093	***	(0.05)
Bait Density 75	-0.95218	**	(0.36)	-1.55423	***	(0.09)	-0.92416	**	(0.36)
Years of Baiting	0.46516	***	(0.07)	0.40909	***	(0.06)	0.46592	***	(0.07)
Period Post-ORV: Sex	--		--	--		--	0.3295		(0.25)
Period Post-ORV: Age	0.23216	***	(0.06)	0.23569	***	(0.06)	0.22559	**	(0.09)
Sex:Age	--		--	--		--	0.06966		(0.07)
Bait Density 75: Years of Baiting	-0.2509	.	(0.14)	--		--	-0.26275	.	(0.14)
Period Post-ORV: Sex: Age	--		--	--		--	0.02942		(0.13)

Significant codes: 0 ‘***’ 0.001 ‘**’ 0.01 ‘*’ 0.05 ‘.’ 0.1 ‘ ’ 1

Table S19: Parameters and estimates of the top competitive models for the individual level generalized additive modeling for skunks based on a rabies virus neutralizing antibody (RVNA) cutoff at 0.5 IU/mL. Oral rabies vaccination (ORV) occurred with Ontario Rabies Vaccine Baits (ONRAB) in West Virginia, USA.

	**Model Name**								
	**m010**			**m007**			**m005**		
**Parameter**	**Estimate (SE)**			**Estimate (SE)**			**Estimate (SE)**		
Intercept	-4.0837	***	(0.73)	-3.7739	***	(0.88)	-4.179	***	(0.77)
Period Post-ORV	1.7263	***	(0.52)	1.3551	.	(0.80)	1.7219	***	(0.52)
Sex	--		--	--		--	0.1465		(0.35)
Age	0.4741	*	(0.20)	0.2393		(0.47)	0.4805	*	(0.20)
Bait Density 75	3.6957		(2.25)	3.6993	.	(2.25)	3.8229	.	(2.27)
Years of Baiting	0.5004	*	(0.23)	0.5055	*	(0.23)	0.5052	*	(0.23)
Period Post-ORV: Sex	--		--	--		--	--		--
Period Post-ORV: Age	--		--	0.2901		(0.52)	--		--
Sex:Age	--		--	--		--	--		--
Bait Density 75: Years of Baiting	-2.1691	*	(0.94)	-2.1721	*	(0.94)	-2.216	*	(0.94)
Period Post-ORV: Sex: Age	--		--	--		--	--		--

Significant codes: 0 ‘***’ 0.001 ‘**’ 0.01 ‘*’ 0.05 ‘.’ 0.1 ‘ ’ 1

Table S20: Model selection table of the individual level generalized additive modeling candidate model set for raccoons based on detection of the tetracycline biomarker. Models are sorted from most parsimonious on top to least parsimonious based on Akaike's information criterion (AICc) corrected for small sample sizes. The model names, degrees of freedom (df), the log likelihood (LL), the AICc, the delta AICc (difference between the model AICc value and the top model), the model likelihood(ModelLik), the AICc weight (relative support for each model), and the cumulative AICc weight (Cum.Wt, representing total cumulative weight for the given model and all models above it) are shown.

Model Name	df	LL	AICc	delta AICc	Cum. Wt
m001	13	-1385.869	2798.671	0.00000	0.9895
m015	12	-1391.500	2807.873	9.20233	0.0099
m004	10	-1396.762	2814.466	15.79584	0.0004
m003	10	-1397.614	2815.819	17.14861	0.0002
m007	9	-1400.805	2820.520	21.84929	0.0000
m018	9	-1402.292	2823.474	24.80366	0.0000
m017	9	-1402.936	2824.426	25.75565	0.0000
m002	10	-1402.841	2826.434	27.76305	0.0000
m021	8	-1406.042	2828.960	30.28945	0.0000
m005	9	-1406.891	2832.480	33.80929	0.0000
m016	9	-1408.013	2834.737	36.06593	0.0000
m010	8	-1410.820	2838.302	39.63156	0.0000
m019	8	-1411.994	2840.649	41.97795	0.0000
m008	9	-1412.299	2842.952	44.28136	0.0000
m024	7	-1415.638	2845.923	47.25217	0.0000
m006	9	-1415.572	2849.619	50.94852	0.0000
m022	8	-1417.045	2850.442	51.77097	0.0000
m009	8	-1419.630	2855.693	57.02211	0.0000
m020	8	-1420.163	2856.774	58.10326	0.0000
m011	8	-1420.386	2857.255	58.58474	0.0000
m014	7	-1423.346	2861.152	62.48139	0.0000
m023	7	-1424.170	2862.750	64.07958	0.0000
m012	7	-1424.475	2863.351	64.68040	0.0000
m025	7	-1424.973	2864.424	65.75385	0.0000
m028	6	-1427.711	2867.894	69.22376	0.0000
m026	6	-1428.693	2869.790	71.11974	0.0000
m013	6	-1444.485	2902.882	104.21098	0.0000
m071	5	-1448.078	2908.072	109.40107	0.0000
m027	5	-1448.206	2908.381	109.71042	0.0000
m072	4	-1451.554	2913.104	114.43338	0.0000
m029	11	-1445.284	2913.864	115.19330	0.0000
m032	8	-1456.063	2929.418	130.74724	0.0000
m031	8	-1458.614	2934.354	135.68354	0.0000
m035	7	-1459.563	2934.411	135.74049	0.0000
m030	8	-1464.142	2945.481	146.80998	0.0000

m033	7	-1467.821	2950.814	152.14377	0.0000
m038	6	-1471.273	2955.715	157.04475	0.0000
m036	7	-1475.070	2965.185	166.51431	0.0000
m034	7	-1475.670	2966.456	167.78583	0.0000
m037	6	-1479.306	2971.713	173.04222	0.0000
m040	5	-1483.645	2978.393	179.72262	0.0000
m039	6	-1482.886	2978.865	180.19441	0.0000
m042	5	-1485.372	2981.835	183.16418	0.0000
m041	5	-1505.874	3022.731	224.06005	0.0000
m073	4	-1509.011	3027.014	228.34370	0.0000
m043	11	-1505.586	3035.287	236.61644	0.0000
m045	8	-1514.783	3047.625	248.95397	0.0000
m046	8	-1517.425	3052.908	254.23784	0.0000
m044	8	-1521.932	3061.922	263.25147	0.0000
m049	7	-1523.122	3062.286	263.61564	0.0000
m047	7	-1525.865	3067.773	269.10199	0.0000
m057	10	-1526.179	3074.446	275.77523	0.0000
m052	6	-1531.446	3076.920	278.24961	0.0000
m048	7	-1530.751	3077.545	278.87431	0.0000
m050	7	-1531.269	3078.582	279.91161	0.0000
m051	6	-1534.721	3083.472	284.80119	0.0000
m059	7	-1536.724	3089.485	290.81459	0.0000
m060	7	-1537.857	3091.751	293.08036	0.0000
m054	5	-1541.201	3094.418	295.74711	0.0000
m053	6	-1541.031	3096.092	297.42102	0.0000
m063	6	-1543.133	3100.289	301.61823	0.0000
m056	5	-1545.313	3102.643	303.97246	0.0000
m058	7	-1543.759	3103.555	304.88407	0.0000
m061	6	-1547.568	3109.160	310.48900	0.0000
m066	5	-1552.723	3117.458	318.78747	0.0000
m062	6	-1552.817	3119.656	320.98530	0.0000
m064	6	-1554.286	3122.596	323.92544	0.0000
m065	5	-1556.642	3125.295	326.62442	0.0000
m055	5	-1560.227	3132.471	333.80050	0.0000
m068	4	-1562.702	3135.404	336.73360	0.0000
m067	5	-1563.682	3139.376	340.70573	0.0000
m074	4	-1565.212	3140.432	341.76095	0.0000
m070	4	-1567.546	3145.094	346.42302	0.0000
m069	4	-1583.566	3177.134	378.46314	0.0000
m075	3	-1588.139	3184.271	385.60072	0.0000

Table S21: Model selection table of the individual level generalized additive modeling candidate model set for skunks based on detection of the tetracycline biomarker. Models are sorted from most parsimonious on top to least parsimonious based on Akaike's information criterion corrected (AICc) for small sample sizes. The model names, degrees of freedom (df), the log likelihood (LL), the AICc, the delta AICc (difference between the model AICc value and the top model), the model likelihood(ModelLik), the AICc weight (relative support for each model), and the cumulative AICc weight (Cum.Wt, representing total cumulative weight for the given model and all models above it) are shown.

Model Name	df	LL	AICc	delta AICc	Cum. Wt
m021	6	-69.482	151.391	0.00000	0.2216
m018	7	-69.147	152.865	1.47410	0.1060
m007	7	-69.241	153.054	1.66306	0.0965
m028	4	-72.553	153.308	1.91687	0.0850
m014	4	-72.335	154.237	2.84544	0.0534
m004	8	-68.935	154.609	3.21814	0.0443
m025	5	-72.160	154.623	3.23192	0.0440
m024	5	-72.425	155.153	3.76172	0.0338
m072	3	-74.592	155.304	3.91272	0.0313
m011	5	-71.967	155.612	4.22130	0.0268
m027	4	-73.816	155.834	4.44298	0.0240
m022	6	-71.844	156.115	4.72421	0.0209
m026	4	-74.093	156.388	4.99714	0.0182
m019	6	-72.028	156.484	5.09246	0.0174
m010	6	-72.201	156.829	5.43780	0.0146
m016	7	-71.175	156.921	5.53009	0.0140
m009	5	-73.196	157.014	5.62238	0.0133
m071	4	-74.406	157.014	5.62303	0.0133
m023	5	-73.368	157.039	5.64748	0.0132
m020	6	-72.511	157.449	6.05776	0.0107
m013	5	-73.656	157.616	6.22489	0.0099
m035	5	-73.668	157.641	6.24961	0.0097
m008	7	-71.660	157.892	6.50072	0.0086
m005	6	-71.831	158.011	6.62019	0.0081
m017	7	-71.737	158.046	6.65459	0.0080
m012	5	-73.894	158.092	6.70056	0.0078
m015	10	-68.581	158.303	6.91174	0.0070
m002	8	-70.998	158.734	7.34318	0.0056
m032	6	-73.275	158.978	7.58663	0.0050
m003	7	-71.548	159.061	7.67021	0.0048
m006	7	-72.354	159.281	7.88950	0.0043
m042	3	-76.999	160.120	8.72885	0.0028
m001	11	-68.377	160.130	8.73875	0.0028
m073	2	-77.940	160.871	9.47985	0.0019
m040	3	-77.507	161.169	9.77826	0.0017

m039	4	-76.524	161.249	9.85798	0.0016
m041	3	-77.603	161.328	9.93666	0.0015
m038	4	-76.577	161.357	9.96540	0.0015
m037	4	-76.865	161.932	10.54079	0.0011
m036	5	-76.062	162.428	11.03717	0.0009
m033	5	-76.122	162.548	11.15672	0.0008
m034	5	-76.171	162.646	11.25455	0.0008
m030	6	-75.434	163.295	11.90408	0.0006
m031	6	-75.694	163.815	12.42427	0.0004
m029	9	-72.738	164.405	13.01358	0.0003
m075	2	-84.988	175.027	23.63560	0.0000
m070	3	-84.502	175.739	24.34778	0.0000
m063	5	-82.519	175.834	24.44305	0.0000
m069	3	-84.713	176.159	24.76793	0.0000
m074	3	-84.537	176.179	24.78767	0.0000
m056	4	-83.870	176.547	25.15549	0.0000
m068	3	-84.950	176.848	25.45639	0.0000
m049	6	-81.934	176.853	25.46181	0.0000
m067	4	-84.302	176.914	25.52275	0.0000
m064	4	-83.413	176.925	25.53392	0.0000
m060	5	-82.278	176.980	25.58910	0.0000
m055	4	-84.275	177.376	25.98532	0.0000
m066	4	-84.514	177.787	26.39541	0.0000
m053	5	-83.695	177.828	26.43640	0.0000
m062	5	-83.615	177.850	26.45912	0.0000
m050	5	-82.811	177.858	26.46702	0.0000
m065	4	-84.691	177.950	26.55841	0.0000
m054	4	-84.533	178.125	26.73419	0.0000
m046	7	-81.725	178.141	26.74952	0.0000
m052	5	-83.870	178.647	27.25544	0.0000
m058	6	-83.170	178.810	27.41910	0.0000
m061	5	-84.320	178.954	27.56240	0.0000
m059	5	-83.419	178.986	27.59510	0.0000
m048	6	-83.149	179.066	27.67447	0.0000
m051	5	-84.284	179.298	27.90639	0.0000
m044	7	-82.475	179.630	28.23897	0.0000
m047	6	-83.704	179.943	28.55210	0.0000
m045	6	-82.813	179.994	28.60296	0.0000
m057	8	-80.987	180.808	29.41731	0.0000
m043	9	-80.392	181.893	30.50223	0.0000

Table S22: Parameters and estimates of the top competitive models for the individual level generalized additive modeling for raccoons based on detection of the tetracycline biomarker. Oral rabies vaccination (ORV) occurred with Ontario Rabies Vaccine Baits (ONRAB) in West Virginia, USA.

	**Model Name**		
	**m001**		
**Parameter**	**Estimate (SE)**		
Intercept	-1.38308	***	(0.24)
Period Post-ORV	1.03333	***	(0.21)
Sex	-0.01877		(0.21)
Age	-0.16242	**	(0.06)
Bait Density 75	-0.34058		(0.37)
Years of Baiting	0.86856	***	(0.09)
Period Post-ORV: Sex	-0.16857		(0.27)
Period Post-ORV: Age	-0.21252	*	(0.09)
Sex:Age	0.26802	**	(0.08)
Bait Density 75: Years of Baiting	-0.5187	***	(0.16)
Period Post-ORV: Sex: Age	-0.13039		(0.13)

Significant codes: 0 ‘***’ 0.001 ‘**’ 0.01 ‘*’ 0.05 ‘.’ 0.1 ‘ ’ 1

Table S23: Parameters and estimates of the top competitive models for the individual level generalized additive modeling for skunks based on detection of the tetracycline biomarker. Oral rabies vaccination (ORV) occurred with Ontario Rabies Vaccine Baits (ONRAB) in West Virginia, USA.

	**Model Name**											
	**m021**			**m018**			**m007**			**m028**		
**Parameter**	**Estimate (SE)**			**Estimate (SE)**			**Estimate (SE)**			**Estimate (SE)**		
Intercept	-3.7834	***	(0.95)	-3.9997	***	(1.00)	-3.7480	***	(0.95)	-2.6216	***	(0.64)
Period Post-ORV	1.4374	.	(0.78)	1.4324	.	(0.78)	1.4410	.	(0.78)	--		--
Sex	--		--	0.3634		(0.45)	--		--	--		--
Age	0.3614		(0.41)	0.3891		(0.41)	0.3584		(0.41)	-0.4897	.	(0.26)
Bait Density 75	-3.5811	***	(1.05)	-3.5737	***	(1.06)	-124.8000		(4.7e+7)	-3.433	**	(1.05)
Years of Baiting	0.8036	**	(0.29)	0.7965	**	(0.29)	0.7867	**	(0.29)	0.8006	**	(0.28)
Period Post-ORV: Sex	--		--	--		--	--		--	--		--
Period Post-ORV: Age	-1.3772	*	(0.59)	-1.3606	*	(0.59)	-1.3790	*	(0.59)	--		--
Sex:Age	--		--	--		--	--		--	--		--
Bait Density 75: Years of Baiting	--		--	--		--	40.4800		(1.6e+7)	--		--
Period Post-ORV: Sex: Age	--		--	--		--	--		--	--		--

Significant codes: 0 '***' 0.001 '**' 0.01 '*' 0.05 '.' 0.1 ' ' 1
